# deFuse: An Algorithm for Gene Fusion Discovery in Tumor RNA-Seq Data

**DOI:** 10.1371/journal.pcbi.1001138

**Published:** 2011-05-19

**Authors:** Andrew McPherson, Fereydoun Hormozdiari, Abdalnasser Zayed, Ryan Giuliany, Gavin Ha, Mark G. F. Sun, Malachi Griffith, Alireza Heravi Moussavi, Janine Senz, Nataliya Melnyk, Marina Pacheco, Marco A. Marra, Martin Hirst, Torsten O. Nielsen, S. Cenk Sahinalp, David Huntsman, Sohrab P. Shah

**Affiliations:** 1 Centre for Translational and Applied Genomics, BC Cancer Agency, Vancouver, British Columbia, Canada; 2 School of Computing Science, Simon Fraser University, Burnaby, British Columbia, Canada; 3 Canada's Michael Smith Genome Sciences Centre, BC Cancer Agency, Vancouver, British Columbia, Canada; 4 Department of Pathology and Laboratory Medicine, University of British Columbia, Vancouver, Canada; 5 Department of Molecular Oncology, BC Cancer Agency, Vancouver, British Columbia, Canada; Accelrys, United States of America

## Abstract

Gene fusions created by somatic genomic rearrangements are known to play an important role in the onset and development of some cancers, such as lymphomas and sarcomas. RNA-Seq (whole transcriptome shotgun sequencing) is proving to be a useful tool for the discovery of novel gene fusions in cancer transcriptomes. However, algorithmic methods for the discovery of gene fusions using RNA-Seq data remain underdeveloped. We have developed deFuse, a novel computational method for fusion discovery in tumor RNA-Seq data. Unlike existing methods that use only unique best-hit alignments and consider only fusion boundaries at the ends of known exons, deFuse considers all alignments and all possible locations for fusion boundaries. As a result, deFuse is able to identify fusion sequences with demonstrably better sensitivity than previous approaches. To increase the specificity of our approach, we curated a list of 60 true positive and 61 true negative fusion sequences (as confirmed by RT-PCR), and have trained an adaboost classifier on 11 novel features of the sequence data. The resulting classifier has an estimated value of 0.91 for the area under the ROC curve. We have used deFuse to discover gene fusions in 40 ovarian tumor samples, one ovarian cancer cell line, and three sarcoma samples. We report herein the first gene fusions discovered in ovarian cancer. We conclude that gene fusions are not infrequent events in ovarian cancer and that these events have the potential to substantially alter the expression patterns of the genes involved; gene fusions should therefore be considered in efforts to comprehensively characterize the mutational profiles of ovarian cancer transcriptomes.

## Author Summary

Genome rearrangements and associated gene fusions are known to be important oncogenic events in some cancers. We have developed a novel computational method called deFuse for detecting gene fusions in RNA-Seq data and have applied it to the discovery of novel gene fusions in sarcoma and ovarian tumors. We assessed the accuracy of our method and found that deFuse produces substantially better sensitivity and specificity than two other published methods. We have also developed a set of 60 positive and 61 negative examples that will be useful for accurate identification of gene fusions in future RNA-Seq datasets. We have trained a classifier on 11 novel features of the 121 examples, and show that the classifier is able to accurately identify real gene fusions. The 45 gene fusions reported in this study represent the first ovarian cancer fusions reported, as well as novel sarcoma fusions. By examining the expression patterns of the affected genes, we find that many fusions are predicted to have functional consequences and thus merit experimental followup to determine their clinical relevance.

## Introduction

Gene fusions are known to play an important role in the development of haematalogical disorders and childhood sarcomas, while the recent discovery of ETS gene fusions in prostate cancer [1] has also prompted renewed interest in gene fusions in solid tumors. ETS gene fusions are present in 80% of malignancies of the male genital organs, and as a result these fusions alone are associated with 16% of all cancer morbidity [2]. The discovery of the *EML4*-*ALK* fusion in non-small-cell lung cancer and the *ETV6*-*NTRK3* fusion in human secretory breast carcinoma suggest that gene fusions are also recurrent at low levels in other solid tumor types [3], [4]. The discovery of such rare but recurrent gene fusions may be of significant clinical benefit where they provide the potential for targeted therapy.

Gene fusions are thought to arise predominantly from double stranded DNA breakages followed by a DNA repair error [2], [5]. Promoter exchanges are one class of gene fusions, characterized by the replacement of an oncogene's regulatory regions with those of another gene, resulting in deregulation of transcription of the oncogene. For ETS gene fusions in prostate cancer, the androgen-responsive regulatory elements of *TMPRSS2* drive the expression of the ETS family member to which *TMPRSS2* is fused [1]. Another class of gene fusions leads to the creation of a chimeric protein with biological function distinct from either of the partner genes from which it originated. A classic example is *BCR*-*ABL1*, a chimeric protein that is the defining lesion in chronic myelogenous leukaemia (CML), and which induces growth factor independence and the inhibition of apoptosis [6].

Large scale, genome-wide efforts to comprehensively identify and characterize genomic rearrangements that lead to gene fusions in human cancers have recently been made possible through next generation sequencing technologies. These technologies provide a deeper level of sequencing than is possible by cytogenetic and Sanger sequencing methods and are poised to reveal a more detailed understanding of the extent and nature of genomic rearrangements in cancer. For example, using low-coverage paired end whole genome (gDNA) shotgun sequencing, Stephens et al. [7] reported that the genomes of breast cancer cells harbour many more rearrangements than previously thought, and suggested that this class of somatic mutation needs to be carefully considered when interpreting breast cancer genomes. Using similar experimental and analytical techniques, Campbell et al. [8] profiled tumor evolution in pancreatic cancer patient samples by profiling the pattern of somatic rearrangements found in primary tumors and distant metastases extracted from the same patient.

### Previous work on gene fusion detection from RNA-Seq

Next generation sequencing of cDNA (RNA-Seq or whole transcriptome shotgun sequencing) provides an ideal experimental platform for expressed gene fusion discovery. Analogous to genome sequencing, RNA-Seq enables an unbiased and relatively comprehensive view into tumor transcriptomes, and can provide information about the rarest of transcripts. RNA-Seq targets only expressed sequences from protein coding genes and is thus more focused than whole genome sequencing. Maher et al. [9] demonstrated the capacity of RNA-Seq to find gene fusions in prostate cancer samples. They identified potentially fused gene pairs using discordantly aligned paired end reads, and also identified potential fusion splices by mining end sequences for alignments to all possible pairings of exons of the potentially fused gene pairs. A study of the melanoma transcriptome by Berger et al. (2010) used many of the same principles. Another recently developed method called FusionSeq identifies gene fusions from discordant alignments, and uses a variety of novel filters and quality metrics to discriminate real fusions from sequencing and alignment artifacts [10]. FusionSeq has been used to identify fusions in prostate tumor samples and cell lines [10], [11]. While the methods used for these studies are capable of identifying genuine gene fusions, many challenges and limitations remain in the analysis of RNA-Seq data. For example, the aforementioned studies only considered reads that align uniquely to the genome. However, errors in next generation sequencing together with homologous and repetitive sequences shared between genes often produce ambiguous alignments of the short reads generated in RNA-Seq experiments. While resolving the ‘correct’ placement of these reads is often not possible, we propose that ambiguously-aligning reads provide important evidence of real gene fusions, and therefore should be leveraged by analysis methods.

Sequence reads that align across a gene fusion boundary (so-called *split reads*) are a strong source of evidence for gene fusions in paired-end RNA-Seq data. Hu et al. [12] propose a strategy centered on the ability to identify split reads called PERAlign: the method uses split read aligner MapSplice [13] to identify single end reads split by fusion boundaries, and then verifies those fusion boundaries using a probabilistic model to infer the alignment of discordantly aligning pairs. As described below, our method also combines the complementary sources of split reads and discordant reads, but we show using real patient data that discordant read analysis *followed by* split read analysis is considerably more sensitive for gene fusion discovery than the reverse procedure described by Hu et al.

### The deFuse method

With the goal of resolving the limitations described above and therefore providing a more accurate method for detecting gene fusions from RNA-Seq, we developed a novel algorithm called *deFuse*. The central idea behind deFuse is to guide a dynamic programming-based split read analysis with discordant paired end alignments. This is in contrast to PERAlign, which uses discordant paired end alignments to verify the results of a split read analysis. Furthermore, unlike previous approaches, we do not discard paired end reads that align ambiguously, but instead consider all alignments for each read, and attempt to resolve the most likely alignment position for each read. We show that using ambiguously-aligning reads results in an increased amount of evidence for predicted gene fusions and an increase in the number of relevant gene fusions predicted. In addition, our method is not limited to finding gene fusions with boundaries between known exons, and therefore can identify fusion boundaries in the middle of exons or involving intronic or intergenic sequences. Finally, the method attempts to provide a number of confidence measures to estimate the validity of each prediction.

## Methods

### Ethics statement

We obtained three sarcomas and 40 ovarian carcinomas from the OvCaRe (Ovarian Cancer Research) frozen tumor bank. Patients provided written informed consent for research using these tumor samples before undergoing surgery, and the consent form acknowledged that a loss of confidentiality could occur through the use of samples for research. Separate approval from the hospital's institutional review board was obtained to permit the use of these samples for RNA-sequencing experiments.

### Data sets

#### Patient tumor samples from the OvCaRe tumor bank

We interrogated the transcriptomes of a cell line derived from a serous borderline tumor, in addition to three sarcomas and 40 ovarian carcinomas obtained from the OvCaRe (Ovarian Cancer Research) frozen tumor bank. Pathology review, sample preparation, RNA extraction, RNA-Seq library construction and RNA-Seq sequence data generation using Illumina GA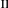 were performed as previously described [14], [15]. The RNA-Seq datasets used in this study are listed in Table 1, which provides a summary-level description of each sample. For each case we list data acquisition statistics, the tumor type and subtype and the number of predictions made by deFuse.

**Table 1:** Summary of RNA-Seq and fusion analysis.

Case	Type	Reads (Millions)	Read Length	Fragment Mean	Fragment Std. Dev.	Total Fusions	In-frame	Inter-chr.	Intra-chr.	Read-through	Inversion	Eversion	Deletion
SBOT	LGS	28	36–42	210	38	24	2	2	22	17	3	1	1
CCC1	CCC	18	50	282	36	49	6	10	39	30	1	3	5
CCC2	CCC	38	50	198	29	27	0	5	22	18	3	0	1
CCC3	CCC	37	50	209	27	34	2	6	28	22	3	0	3
CCC4	CCC	20	50	249	41	55	7	7	48	33	7	8	0
CCC5	CCC	32	36–42	245	36	26	1	6	20	17	2	0	1
CCC6	CCC	32	36–42	234	38	14	3	0	14	10	1	1	2
CCC7	CCC	19	50	259	39	48	4	12	36	21	4	6	5
CCC8	CCC	39	36–42	242	38	41	7	12	29	15	2	10	2
CCC9	CCC	38	50	265	41	62	13	10	52	35	5	6	6
CCC10	CCC	37	50	278	38	97	10	12	85	75	5	1	4
CCC11	CCC	53	36–42	259	39	64	2	24	40	33	4	2	1
CCC12	CCC	36	36–42	244	31	40	7	10	30	18	8	4	0
CCC13	CCC	31	50	263	35	74	10	15	59	49	3	6	1
CCC14	CCC	40	50	250	39	82	8	13	69	56	6	4	3
CCC15	CCC	40	50	189	29	53	6	16	37	19	5	9	4
CCC16	CCC	41	50	229	27	80	2	16	64	46	5	8	5
EMD1	EMD	32	36–50	187	35	62	5	9	53	37	9	1	6
EMD2	EMD	30	42–50	208	33	64	7	2	62	50	6	5	1
EMD3	EMD	33	50	227	31	40	4	6	34	30	4	0	0
EMD4	EMD	38	50	242	33	58	7	3	55	41	8	3	3
EMD5	EMD	39	50–75	244	29	49	6	3	46	38	4	2	2
EMD6	EMD	39	50	246	34	85	11	12	73	45	11	11	6
EMD7	EMD	25	42–50	211	33	23	4	3	20	15	3	1	1
EMD8	EMD	30	50–75	189	31	51	7	3	48	40	2	3	3
GRC1	GRC	58	36–50	206	39	105	14	10	95	78	8	3	6
GRC2	GRC	74	36–42	183	39	95	5	15	80	60	12	0	8
GRC3	GRC	31	36–42	196	37	38	3	5	33	29	3	1	0
GRC4	GRC	34	36–42	172	34	46	5	7	39	27	7	1	4
GRC5	GRC	41	50–75	247	31	101	9	8	93	71	16	0	6
HGS1	HGS	39	50	241	37	73	6	9	64	51	8	2	3
HGS2	HGS	29	50	278	38	75	12	8	67	58	5	1	3
HGS3	HGS	26	37–42	211	34	80	7	15	65	59	3	2	1
HGS4	HGS	30	36–42	209	33	54	3	11	43	20	8	10	5
HGS5	HGS	33	50	220	25	92	7	11	81	65	7	6	3
LGS1	LGS	35	50	242	26	47	8	3	44	34	9	0	1
MUC1	MUC	42	36–50	208	30	66	8	11	55	44	6	3	2
MUC2	MUC	33	36	224	31	61	9	11	50	37	10	1	2
SCH1	SCH	24	50–75	210	30	43	3	11	32	27	5	0	0
SCH2	SCH	35	36–50	201	31	46	0	6	40	34	4	1	1
YKS1	YKS	46	50	249	27	44	6	5	39	34	3	1	1
YKS2	YKS	40	50	252	31	49	5	11	38	32	3	1	2
SARC1	EPS	19	50	263	35	39	1	6	33	27	3	2	1
SARC2	EPS	28	36–50	333	36	69	10	10	59	51	6	0	2
SARC3	IGMS	17	50	233	33	15	2	4	11	10	0	1	0

Summary of RNA-Seq statistics and fusion predictions across all samples. LGS: Low Grade Serous, HGS: High Grade Serous, CCC: Clear cell carcinoma, EMD: Endometrioid tumor, MUC: Mucinous tumor, YKS: Yolk sac tumor, GRC: Granulosa cell tumor, SCH: Small cell hypercalemic, EPS: Epithelioid Sarcoma, IGMS: intermediate grade myofibroblastic sarcoma.

#### Published data sets

In addition to our internally generated data, we tested deFuse using published paired end RNA-Seq data sets known to contain gene fusions. These datasets were used as positive controls in the evaluation of deFuse. We used the NCI-H660 prostate cell line from the FusionSeq website http://info.gersteinlab.org/FusionSeq_Test_Datasets) known to harbour a TMPRSS2-ERG fusion. In addition, we downloaded the datasets derived from 13 melanoma samples and cell lines and one chronic myelogenous leukemia (CML) cell line K-562 described in Berger et al. [16]. Datasets were obtained from the Short Read Archive (http://www.ncbi.nlm.nih.gov/sra) under submission number SRA009053. As described in Berger et al. [16], the CML cell line harbours three previously described gene fusions including *BCR*-*ABL1*, and the melanoma data harbours 11 gene fusions.

### The deFuse algorithm

In this section, we describe the deFuse algorithm. We begin by defining essential terms. We define a *fragment* as a size selected cDNA sequence (usually approximately 250 bp) during RNA-Seq library construction. We define a *read* as a sequenced end of a fragment (usually approximately 50 bp). We define *paired ends* as the pair of reads sequenced from the ends of the same fragment. The *insert sequence* is the portion in the middle of the fragment that is not sequenced. A *fusion boundary* is the precise, nucleotide-level genomic coordinate that defines the breakpoint on either side of the gene fusion. We define *spanning reads* as paired ends that harbour a fusion boundary in the insert sequence, whereas a *split read* harbours a fusion boundary in the read itself. A *discordant alignment* is produced by spanning reads of a fragment with each end aligning to a different gene, whereas a split read will often produce a single end anchored alignment for which one end aligns to one gene and the other end does not align.

**Figure 1:**
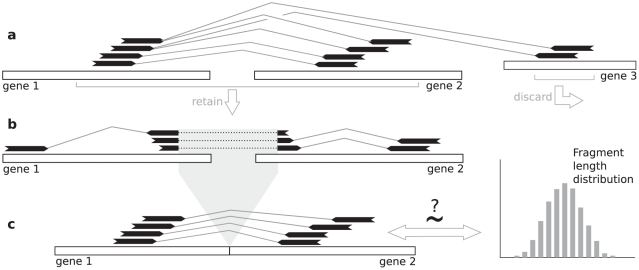
The deFuse gene fusion discovery method. **a**) Discordant alignments are clustered based on the likelihood that those alignments were produced by reads spanning the same fusion boundary. Ambiguous alignments are resolved by selecting the most likely set of fusion events, and the most likely assignment of paired end reads to those events, and the remaining alignments are discarded. **b**) Paired end reads with an alignment for which one end aligns near the approximate fusion boundary are mined for split alignments of the other end of the read. **c**) The predicted fusion boundary is used to calculate the fragment lengths for each spanning paired end read. These fragment lengths are tested for the hypothesis that they were drawn at random from the fragment length distribution.

With these definitions in hand we will now describe how deFuse predicts gene fusions by searching RNA-Seq data for fragments that harbour fusion boundaries. As mentioned previously, the problem of identifying the true genomic origin of a set of RNA-Seq reads is confounded by several factors, and as a result, a proportion of the RNA-Seq reads will have ambiguous alignments to the genome. The deFuse method, outlined schematically in Figure 1, combines an approach for resolving the actual alignment location of ambiguously aligning spanning reads with a dynamic programming based split read analysis for resolving the nucleotide level fusion boundary with high sensitivity. A novel confidence measure is provided based on the degree of corroboration of evidence supporting the prediction.

The method consists of four main steps. The first step is alignment of paired end reads to a reference comprised of the sequences that are expected to exist in the sample, with all relevant alignments considered. We use spliced and unspliced gene sequences as a reference because we have found that fusion genes often produce splice variants that express intronic sequences, and that some of those splice variants are biologically relevant (unpublished data). We define two necessary conditions for considering discordant alignments to have originated from reads spanning the same fusion boundary and use these conditions to cluster discordant alignments representing the same fusion event. The second step resolves ambiguous discordant alignments by selecting the most likely set of fusion events, and the most likely assignment of spanning reads to those events (Figure 1a). The third step is a targeted search for split reads using a dynamic programming based solution to resolve the nucleotide level fusion boundary of each event (Figure 1b). The forth step involves a test for the corroboration of the spanning and split read evidence. For each spanning read, we calculate the putative length of the fragment that generated that paired end read given the fusion boundary predicted by the split reads. The resulting set of fragment lengths is used to test the hypothesis that those fragments were generated by the inferred fragment length distribution (Figure 1c). Finally, we compute a set of quantitative features, and use an adaboost classifier to discriminate between real gene fusions and artifacts of the sequencing and alignment process.

#### Conditions for considering discordant alignments to have originated from reads spanning the same fusion boundary

deFuse begins with a search for spanning reads as evidence of gene fusion events. We describe two necessary conditions for considering two discordant alignments to have originated from reads spanning the same fusion boundary.

The size selection step of the RNA-Seq library construction protocol results in a collection of cDNA fragments with lengths that we approximate with the inferred fragment length distribution 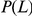 derived from concordant alignments [16], [17]. We restrict our analysis to consider only the most probable range of fragment lengths 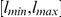 where 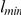 and 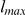 are the 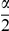 and 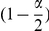-percentiles of 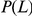 respectively. The value 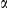 represents the proportion of paired end reads that are not guaranteed by the algorithm to be assigned to the correct fusion event.

**Figure 2:**
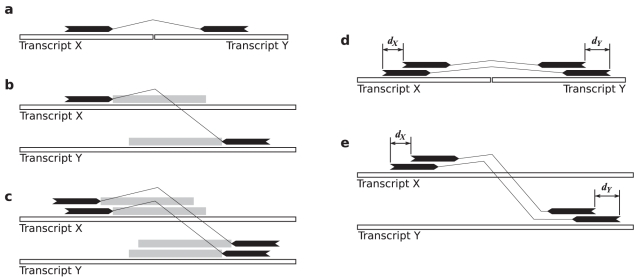
Conditions for considering two paired end reads to have originated from the same fusion transcript. **a**) Fusion transcript X-Y supported by a paired end read spanning the fusion boundary. **b**) Discordant paired end reads represent reads potentially spanning a fusion boundary. Each discordant alignment suggests fusion boundaries in the regions adjacent to the alignments in each transcript. The *fusion boundary region*, shown in gray, is the region in which we expect a fusion boundary to occur. **c**) The *overlapping boundary region condition* is the condition that the fusion boundary regions in each transcript must overlap. **d**) The difference between the fragment lengths of two paired end reads spanning a fusion boundary is 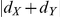. **e**) The *similar fragment length condition* is the constraint that 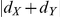 must be no more than 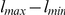.

By definition a spanning read harbours a fusion boundary in the insert sequence (Figure 2a). Given a discordant alignment of a spanning read, the insert sequence of the corresponding fragment should align downstream of the alignment of each end in each gene (Figure 2b). We call the region in the transcript to which the insert sequence should align as the *fusion boundary region* since it represents the region in the transcript where the fusion boundary is expected to exist. Given read length 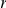, the insert sequence has maximum length 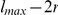, thus the fusion boundary region has length 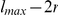.

We define the *overlapping boundary region condition* 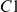 as the condition that the fusion boundary regions for two paired end alignments must overlap in each transcript (Figure 2c). The overlapping boundary region condition ensures that there exists a valid location for the fusion boundary in each transcript that would simultaneously explain both paired end alignments.

Given two reads spanning the same fusion boundary, the difference between the fragment lengths of those reads can be calculated as 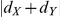 where 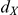 and 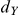 are given by Figure 2d. The implied fragment length difference for two discordant alignments can be calculated similarly as shown in Figure 2e. We define the *similar fragment length condition* 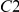 as the constraint that 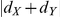 must be no more than 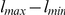 for us to consider two paired end reads to have originated from the same fusion transcript. A more rigorous definition and probabilistic motivation for the two conditions is given in Supplementary Methods (Text S1).

#### Assigning a unique discordant alignment to each spanning read

The utility of an ambiguously aligned read depends on our ability to select the correct alignment for that read based on the greater context of all paired end alignments in the RNA-Seq dataset. Given that we are considering alignments to spliced and unspliced gene sequences, ambiguous alignments will result from homology between genes and also from the redundant representation of the same exon multiple times for multiple splice variants of the same gene. The true alignment for each read must be inferred, as it will be used to identify, for the first situation, the correct pair of genes involved in the fusion, and for the second situation, the correct pair of splice variants of those genes.

Define a *valid cluster* as a set of discordant alignments for which every two paired end alignments in that set satisfy the overlapping boundary region condition 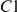 and the similar fragment length condition 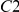. Each valid cluster represents a potential fusion event implied by a set of discordant alignments. A paired end read will be a member of multiple valid clusters as a result of homology between genes, exon redundancy between transcripts, and the multiplicity of valid clusterings. Fusion events are rare when compared with the event of detecting a discordant paired end read given the existence of a fusion event that would generate that read. Thus we seek an assignment of each paired end read to a single fusion event (valid cluster) that minimizes the number of fusion events. The resulting solution, first described by Hormozdiari et al. in the context of genomic structural variation [18], is termed the maximum parsimony solution.

Computation of the maximum parsimony solution is NP-Hard by reduction to the set cover problem as shown by Hormozdiari et al. [18]. Similar to Hormozdiari et al., we compute the maximum parsimony solution using a modified version of the greedy algorithm for solving set cover with approximation factor 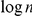. Reads are initially set as *unassigned* and valid clusters are initially set as *unselected*. At each step the algorithm *selects*, from the set of unselected valid clusters, the cluster containing the largest number of unassigned reads, with ties broken randomly. Each unassigned read in the newly selected cluster is *assigned* to that cluster. The algorithm continues, selecting clusters and assigning reads, until all reads have been assigned to clusters. The algorithm will produce an equivalent solution to the maximum parsimony problem when considering only *maximal* valid clusters, rather than all valid clusters (which are exponential in number). Therefore we first calculate the maximal valid clusters (see Supplementary Methods, Text S1) and then apply the algorithm to only the maximal valid clusters.

#### Split read boundary sequence prediction

**Figure 3:**
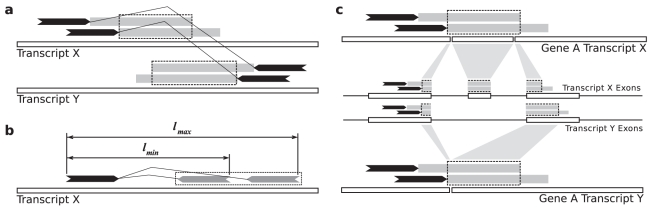
Searching for candidate split reads. **a**) Approximate fusion boundaries, shown as dashed rectangles, are the intersection of fusion boundary regions for discordant alignments supporting a potential fusion. **b**) The mate alignment region, shown as a dashed rectangle, is the union of possible alignment locations for the other end of a single end anchored alignment. **c**) The approximate fusion boundary in transcript 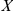 is projected into transcript 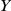 by remapping the start of the approximate fusion boundary from 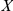, to the genome, to 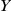.

Given fusion events nominated by spanning reads, deFuse does a targeted split read analysis to predict nucleotide level fusion boundaries. For a cluster of discordant alignments, the *approximate fusion boundary* is the intersection of the fusion boundary regions of the discordant alignments in that cluster (Figure 3a). A *candidate split read* is a read for which one end is anchored near to an approximate fusion boundary, such that the other end of the read could potentially align to the approximate fusion boundary. Reads with discordant and single end anchored alignments are considered as candidate split reads; however, reads with concordant alignments are not considered.

Given the alignment of one end of a read, we define the *mate alignment region* as the region in which we would expect the other end of the read to align for the pair of alignments to be considered concordant. The mate alignment region is calculated as the union of all possible concordant alignments of the other end of the read given a fragment length in the range 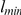 to 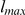 (Figure 3b). A read with a mate alignment region that intersects the approximate fusion boundary of a cluster is considered a candidate split read for that cluster.

The maximum parsimony solution will nominate fusions such that the average number of spanning reads per fusion is maximized. However, it is possible that the selected transcript variants do not maximize both spanning and split read evidence. Thus, when searching for candidate split reads, it is necessary to search across all relevant transcripts. In addition to calculating the approximate fusion boundary in the transcript variants proposed by the maximum parsimony solution, we also project those approximate fusion boundaries onto other transcript variants of the same gene. The approximate fusion boundary in transcript variant 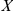 of gene 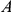 is projected onto transcript 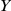 of gene 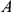 by remapping the start of the approximate fusion boundary from 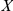 to the genome, and then from the genome to 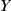 (Figure 3c). The start of the approximate fusion boundary is the end closest to the discordant alignments. Candidate split reads are then found by searching for mate alignment regions that intersect the approximate fusion boundaries in any of the transcript variants.

The split read analysis proceeds by aligning candidate split reads to approximate fusion boundaries. We first align a candidate split read to the two approximate fusion boundaries in the two fused transcripts, then combine the two alignments in a way that maximizes the combined alignment score. Let 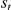 be the end sequence of a candidate split read expected to be split by a fusion boundary and let 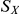 and 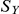 be the sequences of the approximate fusion boundary in transcripts 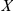 and 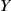, where a fusion between 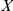 and 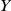 has been nominated by spanning read evidence.

We start by aligning 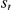 to 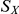 using dynamic programming based local alignment and penalizing initial gaps in the end sequence, and then repeat with the reverse of 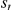 and the reverse of 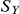 (see Supplementary Methods, Text S1). Let 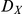 and 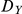 be the matrices produced by aligning 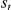 to 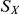 and 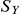. A split in the alignment is represented by the triple 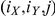 where 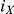 and 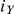 are the nucleotide level position of the fusion boundary in 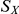 and 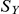, and 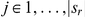 is the position of the fusion boundary in the read sequence with 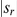 defined as the length of 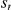. All splits 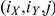 with maximum score can be calculated in 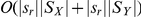 by first finding 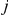 as follows:
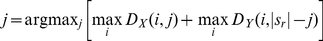
(1)and then finding 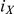 and 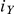 as:
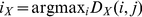
(2)

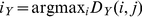
(3)


With this method, finding all splits with maximum score does not necessitate backtracking through a dynamic programming matrix (which is a worst case exponential operation). Additionally, we add the constraint that 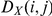 and 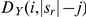 must surpass a threshold score 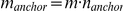, where 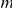 is a score for each matched nucleotide and 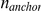 is the minimum number of nucleotides of a potential split read that must align to 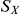 at one end of the read and 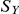 at the other end. Thresholding 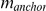 will prevent us from having to consider the case where the majority of a read aligns to 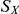 whereas the terminal nucleotide matches erroneously to many locations in 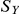, and visa versa.

Multiple splits 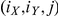 that produce the same maximum score occur for two reasons. Multiple splits with different values for 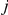 often imply sequence similarity between the regions on each side of the fusion boundary. As a result the subsequence of 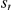 that represents the fusion boundary will align equally well to 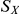 or 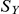. Given a single value for 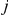, multiple values for 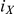 or 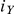 are often the result of one end of the split read aligning to many places because that end is small enough that no unique alignment exist. For the aforementioned situations, the only way to resolve the the true split alignment is to consider the split alignments in the context of the alignments of other candidate split reads.

We resolve the problem of multiple split alignments as follows. We first cluster together split alignments that corroborate the same fusion boundaries 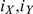. In the unlikely even that a single read produces multiple alignments with the same 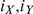 we select the one with the maximum score. For each split alignment in each cluster we calculate the anchoring score 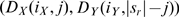 and select the cluster that maximizes the sum of the anchoring scores across all split alignments in that cluster. Maximizing the sum of the anchoring scores has two effects: it attempts to ensure that the fusion boundary is centered in the middle of the reads where multiple fusion boundaries are possible, and it prevents a number of spurious alignments anchored only by a few nucleotides from eclipsing a more interesting fusion boundary prediction.

#### Corroborating spanning read and split read evidence

To test the corroboration between spanning read and split read evidence, we first use the fusion boundary predicted by the split reads to calculate the inferred fragment lengths 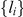 of the 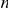 spanning reads that support the fusion prediction. To test the hypothesis that these 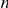 fragment lengths were drawn at random from the same distribution that generated the fragment lengths of concordant paired end reads, we model 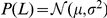 and use a z-test to test the hypothesis that the set of spanning fragment lengths was generated by 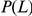. A dependence between the fragment lengths of reads spanning the same fusion boundary means that the sample variance of the set 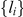 includes a covariance term. The sample variance of 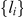 can be calculated as described in Supplementary Methods (Text S1). We use a z-test to calculate the p-value for the hypothesis that the set 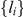 was generated by the distribution 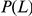. We call this value the corroboration p-value and use it to discriminate between true and false positives.

### Alignment parameters used for this study

To classify paired end reads as concordant we aligned reads to spliced genes, the genome, and UniGene sequences using *bowtie* [19] in paired end mode. We also aligned reads to spliced and unspliced genes in single end mode with parameters −k 100 −m 100. We classified any paired end read as concordant if both ends aligned to the same gene, regardless of the location of the alignment in that gene. Paired end reads aligning with one or both ends to ribosomal RNA sequences were removed from the analysis as has been done previously [10].

Paired end reads not classified as concordant were classified as discordant. Single end mode alignments of discordant paired end reads were then classified as fully aligned or single end anchored. Fully aligned discordant paired end reads were clustered with 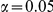 and the maximum parsimony solution was found using the algorithm given above. Split alignments were generated using 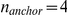, 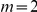, 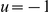, 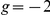 (see Supplementary Methods, Text S1). Finally, a predicted fusion sequence was assembled that included the regions in each gene to which spanning reads aligned, joined together at the fusion boundary predicted by the split reads.

### Annotation of each prediction

Predicted fusion sequences were annotated as open reading frame preserving, 5′ or 3′ UTR exchanges, interchromosomal, inversion, eversion, and between adjacent genes. The translational phase for each coding nucleotide was calculated using the frame column for each exon in the ensembl GTF file. Given nucleotide 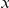 of 5′ gene 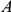 with phase 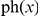 spliced to nucleotide 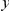 of 3′ gene 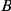 with phase 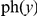, if 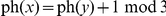, the fusion 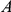
–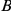 is annotated as open reading frame preserving. Note this method would not detect open reading frames of novel proteins, only those of chimeric proteins that are combination of the protein sequences of the fused genes. Results were also annotated for their position (UTR, exonic, intronic, coding, upstream, downstream) within each gene.

### Classification of predictions as real fusions or false positives

We computed a set of features to better characterize our predicted fusions. The features were calculated for each fusion prediction with the aim of discriminating between true and false positives. We initially lacked a set of positive and negative controls that would have been necessary for a principled machine learning based classification method. Thus initial validation candidates were identified by thresholding these features at levels we suspected would enrich for real fusions (see Results). Validation was also attempted for suboptimal predictions and 40 randomly chosen predictions in order to establish a set of negative controls.

Once we had performed a significant number of validations, these validations became the training set for a classifier. We calculated the following 11 features for the examples in our training set (detailed descriptions in Supplementary Methods, Text S1):

**Spanning read coverage** Normalized spanning read coverage.

**Split position p-value** P-Value for the hypothesis that the *split position* statistic was calculated from split reads that are evenly distributed across the fusion boundary.

**Minimum split anchor p-value** P-Value for the hypothesis that the *minimum split anchor* statistic was calculated from split reads that are evenly distributed across the fusion boundary.

**Corroboration p-value** P-Value for the hypothesis that the lengths of reads spanning the fusion boundary were drawn from the fragment length distribution.

**Concordant ratio** Proportion of spanning reads supporting a fusion that have a concordant alignment using blat with default parameters.

**Fusion boundary di-nucleotide entropy** Di-nucleotide entropy calculated 40 nt upstream and downstream of the fusion boundary for the predicted sequence, taking the minimum of both values.

**Fusion boundary homology** Number of homologous nucleotides in each gene at the predicted fusion boundary.

**cDNA adjusted percent identity** Maximum adjusted percent identity for the alignments of the predicted sequence to any cDNA.

**Genome adjusted percent identity** Maximum adjusted percent identity for the alignments of the predicted sequence to the genome.

**EST adjusted percent identity** Maximum adjusted percent identity for the alignments of the predicted sequence to any EST.

**EST island adjusted percent identity** Maximum adjusted percent identity for the alignments of the predicted sequence to any EST island.

We then used the ada (2.0–2) package in R (2.11.0) to train an adaboost model using the stochastic gradient boosting algorithm with exponential loss, discrete boosting, and decision stumps as the base classifier [20]. We used conservative regularization (shrinkage parameter 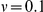) and permitted the algorithm 200 iterations. Adaboost was selected because it would enable us to leverage the weak predictive power of individual features, and would provide a straightforward way of evaluating the predictive power of each feature. Finally, the classifier was used to classify all predictions for our ovarian and sarcoma datasets.

### Implementation, availability and data resources

deFuse is implemented in C++, perl and R. A typical library of 120,000,000 paired end reads completes in approximately 6 hours using a cluster of 100 compute nodes. The human genome (NCBI36) and gene models in GTF format (ensembl 54) were downloaded from Ensembl [21]. EST sequences and spliced EST alignments were downloaded from UCSC [22]. UniGene sequences were downloaded from NCBI [23]. This study focused on the 21295 genes annotated as protein_coding, processed_transcript, IG_C_gene, IG_D_gene, IG_J_gene, and IG_V_gene in the ensembl GTF file (see Table S10 for ensembl IDs for each gene). An unspliced gene sequence is composed of the genomic sequence starting 2 kb upstream of the most upstream exonic nucleotide of that gene's splice variants, to the genomic position 2 kb downstream of the most downstream exonic nucleotide of that gene's splice variants. A spliced gene sequence is composed of the concatenated sequences of each exon of a single splice variant of a gene. Our reference sequences were comprised of 21295 unspliced gene sequences and 46662 spliced gene sequences. All data files are available as part of the deFuse software package at http://compbio.bccrc.ca.

## Results

### Application to ovarian and sarcoma datasets

Fusion sequence predictions were obtained for the 44 datasets as detailed in Methods. This study only considered sequence predictions supported by five or more spanning reads and one or more split reads, though theoretically the limit on the number of spanning reads could be lowered for smaller datasets as was done for the melanoma datasets. The total number of unfiltered predictions at this stage numbered 20,327.

#### Assembling a set of positive and negative controls

Next we assembled a set of positive and negative controls by attempting to validate a selection of predictions potentially representing real fusions, and another set of predictions representing systematic artifacts. To select potential positives, we first used the following set of heuristic filters to enrich for real fusions, producing a subset consisting of 268 predictions.
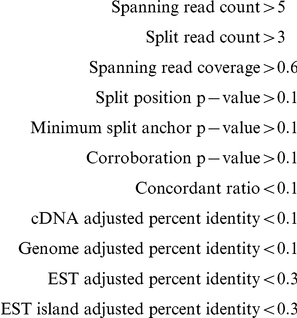



From the 268 filtered predictions we selected 46 predictions, and in doing so attempted to select predictions from libraries with a range of read lengths, such that those predictions covered a large range of values for the spanning and split read counts. Included in this set of 46 predictions were all eight predictions that pass the heuristic filters and involve a cancer associated gene from the cancer gene census (Welcome Trust Sanger Institute Cancer Genome Project web site, http://www.sanger.ac.uk/genetics/CGP). Out of this set of 46 predictions, 42 were successfully validated by RT-PCR (Table S5, Dataset S1).

Next we selected 14 predictions representing potential recurrent artifacts, requiring that each of the 14 fail at least one of our heuristic filters, and also requiring that each was predicted to exist in two or more libraries. None of these predictions validated. Finally, we selected 40 predictions at random from the unfiltered list of 20,327 with the assumption that the majority of them would be negative. Only one of the 40 randomly selected predictions validated as real. In total, 45 predictions were validated by RT-PCR (Table 2). This included 42 predictions from the 46 potential positives, one prediction from the 40 randomly selected fusions, and two more predictions nominated by FusionSeq (see “Comparison to FusionSeq and MapSplice”). Fluorescent in situ hybridization (FISH) assays were attempted for 17 of the 45 PCR validated predictions, with 14 resulting in positive identification of a potentially causative underlying genomic aberration (Table S4, Dataset S2).

**Table 2:** RT-PCR validated novel deFuse predictions.

library	5′ gene	3′ gene	span count	ambig span count	split read count	exon bndry	inter. expr.	prom. exch.	fish valid.	CNV break	split pos. p-value	corrob. p- value	min anchor p-value
CCC1	TYW1	HGSNAT	41	38	11	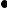	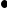 / 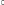	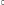	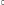		0.92	1	0.65
CCC4	TNS3	PKD1L1	12	0	4	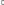	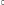 / 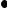	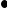			0.82	0.39	0.68
CCC9	RPN2	PMEPA1	48	0	11	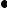	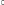 / 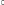	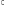	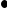		0.83	0.88	0.34
CCC9	TLX3	RANBP17	10	0	5	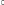	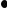 / 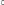	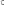			0.51	0.63	0.38
CCC12	ITCH	RALY	59	0	6	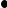	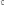 / 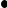	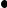		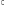 / 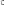	0.99	0.88	0.78
CCC12	MTHFD1	C1orf61	27	8	11	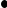	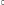 / 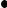	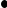	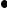	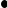 / 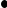	0.53	0.67	0.79
CCC12	YTHDF2	SYTL1	53	0	19	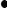	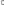 / 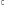	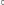		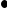 / 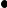	0.75	0.94	0.81
CCC13	PPME1	MRPL48	69	0	22	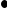	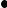 / 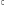	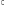			0.48	0.76	0.41
CCC14	EPCAM	DLEC1	27	0	17	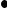	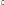 / 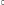	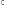	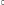		0.98	0.72	0.91
CCC15	AFF4	LAMC3	5	0	3	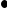	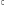 / 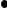	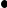		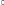 / 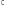	0.64	0.89	0.54
CCC15	ARSB	DMGDH	103	0	87	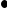	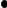 / 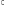	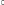		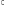 / 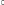	0.92	0.8	0.21
CCC15	KIFC3	CNGB1	14	0	16	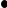	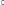 / 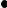	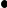		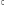 / 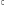	0.36	1	0.45
CCC15	NUMB	ALDH6A1	22	0	12	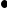	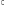 / 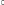	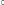		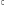 / 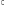	0.98	0.85	0.81
CCC15	PVRL2	LMNA	17	2	7	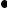	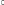 / 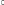	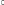	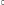	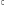 / 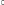	0.39	0.33	0.67
CCC15	SLC38A10	ZCCHC11	12	0	1	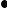	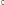 / 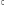	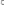		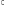 / 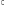	0.28	1	0.29
CCC15	TMEM63A	NRD1	17	0	7	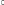	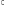 / 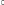	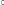	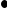	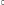 / 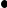	0.57	0.83	0.85
CCC15	UBR4	JMJD2B	27	0	18	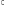	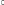 / 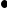	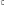	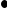	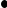 / 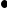	0.5	1	0.56
CCC16	HPS5	APOO	23	3	11	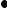	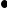 / 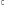	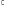	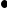		0.67	0.49	0.35
CCC16	PAPOLA	HIP1R	44	0	19	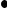	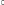 / 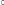	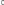	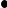		0.89	0.62	0.57
CCC16	PPL	RBKS	10	0	14	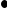	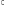 / 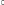	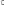	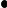		0.81	0.7	0.43
EMD6	BCAS3	ARHGAP15	10	0	4	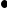	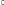 / 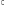	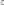	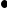	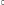 / 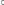	0.42	0.73	0.81
EMD6	CAMK2G	DDX1	9	0	2	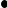	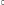 / 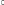	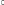		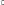 / 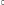	0.28	1	0.46
EMD6	CYB5D2	ANKFY1	6	0	1	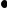	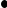 / 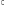	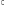		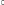 / 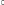	0.65	0.82	0.75
EMD6	EIF4G3	LRRC8D	7	0	4	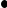	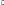 / 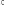	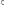		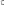 / 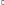	0.19	0.96	0.47
EMD6	ROCK1	CMKLR1	13	0	8	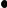	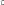 / 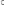	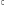	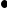	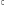 / 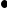	0.62	0.31	0.82
GRC5	FBXO25	BET1L	8	5	3	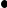	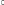 / 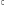	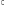			0.86	0.37	0.68
GRC5	PCP4L1	SDHC	7	7	10	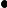	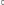 / 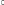	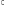			0.71	0.18	0.68
HGS1	CAPNS1	WDR62	7	0	11	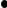	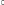 / 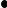	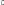			0.55	1	0.85
HGS1	LETM1	USP15	7	1	5	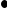	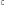 / 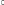	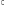			0.95	0.74	0.59
HGS1	RAB6A	USP43	14	9	6	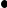	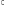 / 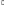	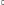	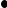		0.45	0.81	0.5
HGS3	ELL	CYLN2	15	0	8	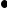	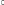 / 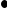	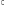		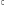 / 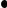	0.85	1	0.56
HGS3	FRYL	SH2D1A	27	0	7	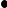	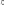 / 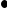	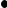	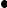	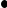 / 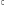	0.9	1	0.62
HGS3	GTF2I	PGPEP1	34	0	3	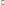	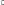 / 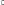	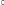		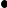 / 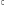	0.15	1	0.3
HGS3	PRR12	FLT3LG	20	0	11	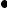	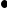 / 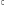	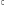		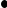 / 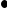	0.72	1	0.24
HGS4	FLNB	VPS8	95	4	51	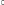	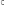 / 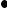	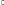	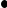	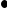 / 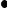	0.8	0.72	0.59
HGS4	LMF1	UMOD	15	0	7	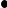	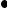 / 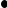	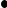		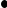 / 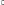	0.88	1	0.4
HGS4	SLC37A1	ABCG1	40	0	14	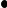	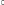 / 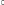	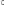		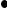 / 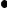	0.83	1	0.42
HGS4	STK3	NPAL2	7	0	3	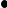	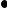 / 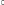	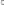		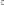 / 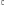	0.69	0.79	0.13
MUC1	ERBB2	PERLD1	25	0	11	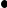	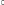 / 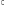	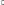		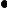 / 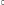	0.84	1	0.75
MUC1	KIAA0355	UQCRC1	10	0	6	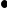	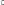 / 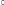	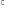		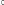 / 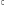	0.82	0.76	0.44
YKS2	C12orf48	MYBPC1	8	0	6	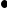	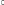 / 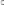	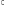		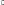 / 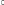	0.63	0.84	0.2
SARC1	CMKLR1	HNF1A	38	0	7	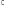	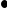 / 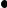	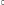			0.72	0.84	0.11
SARC1	ERBB3	CRADD	103	7	41	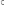	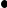 / 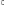	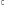	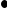		0.87	0.88	0.61
SARC2	SMARCB1	WASF2	16	14	4	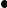	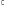 / 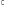	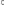		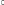 / 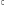	0.38	0.64	0.66
SARC3	RREB1	TFE3	103	0	28	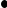	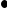 / 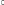	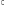	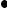	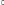 / 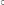	0.93	1	0.3

RNA-Seq evidence, annotation information and validation information is shown for each prediction for which validation by PCR was attempted.

#### Classification of ovarian and sarcoma predictions

**Figure 4:**
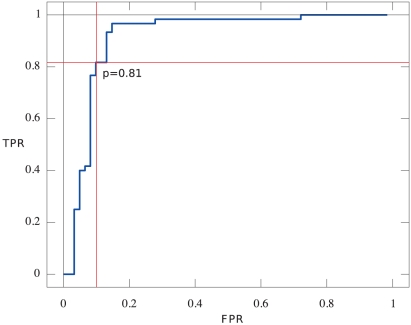
deFuse ROC curve. ROC curve for deFuse annotated with the threshold for the adaboost probability estimate. The threshold corresponds to a false positive rate of 10% and true positive rate of 82%.

**Figure 5:**
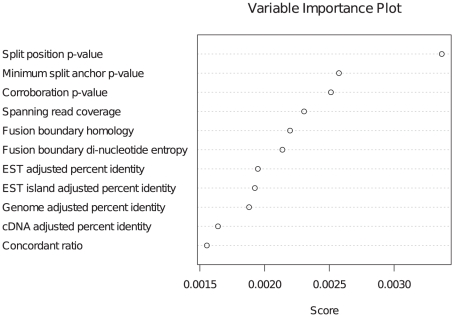
Variable importance plot for deFuse classifier. Relative importance of each of the 11 features used by deFuse classifier.

We were interested in building a classifier that could discriminate between real fusions and false positives. As a training set, we compiled a list of all ovarian and sarcoma fusions for which validation was attempted, and added to this list the 11 melanoma fusions, the three K-562 fusions and the TMPRSS2-ERG fusion in NCI-H660. The resulting dataset contained 60 positive and 61 negative predictions (Table S8). The training set was used to train an adaboost model as described in Methods. Training error for the model was 0.017 representing two negatives misclassified as positives. We used a leave one out method to calculate adaboost probability estimates for each point in the training data. We then used the resulting set of probability estimates to generate an ROC (Figure 4) and calculated the conditional AUC from the ROC as 0.91. Given a target false positive rate of 10%, we calculated the estimated true positive rate as 82%, and a threshold of 0.81 on the probability estimate to achieve that target. Finally we identified the three most significant features as the two p-values calculated for the split alignment positions and the corroboration p-value (Figure 5).

Next we used the adaboost model to classify all remaining ovarian and sarcoma predictions to produce a final set of predictions for the ovarian and sarcoma datasets, thresholding the probability estimates produced by the adaboost model at 0.81. In total we predicted 2,540 gene fusions across all RNA-Seq datasets (Table S1). The vast majority of the 2,540 events, 1,658 in total, were predicted to involve adjacent genes and were not predicted to be the result of an underlying inversion or eversion. For these events, a skipped transcription stop site or alternative transcription start site is an alternative explanation to that of an underlying genomic deletion. Of the remaining 882 events, 394 were inter-chromosomal and 488 were intra-chromosomal. The intra-chromosomal events can be further subdivided into 240 inversions, 131 eversions and 117 deletions effecting non-adjacent genes (Table 1).

### deFuse has higher sensitivity and specificity than competing methods

#### Comparison to FusionSeq and MapSplice

We analyzed CCC15, CCC16 and EMD6 with MapSplice version 1.14.1 and FusionSeq version 0.6.1 in order to compare the sensitivity of these methods with that of deFuse (Supplementary Methods, Text S1, Dataset S3, Dataset S4). These cases were chosen because they ranked highest with respect to the number of validated predictions, with seven, three and four validated predictions respectively. FusionSeq successfully identified ten of a potential 14 validated deFuse predictions in CCC15, CCC16 and EMD6 (Table 2), whereas MapSplice did not recover any of these fusions. We suspected that MapSplice might perform better on RNA-Seq data with longer read lengths. Thus we also used MapSplice to predict fusions using the 75mer reads from GRC5. GRC5 was chosen because it is the only case with 75mers and validated deFuse fusion predictions. MapSplice successfully identified the sequences of both validated fusions in GRC5.

We also attempted to establish whether FusionSeq and MapSplice could identify real fusions in our data that deFuse should have been able to identify, but did not. To this end, we identified all MapSplice and FusionSeq predictions for which there were no corresponding deFuse predictions in the set of initial predictions produced by the heuristic filters. We also removed MapSplice and FusionSeq predictions that did not involve ensembl annotated genes because it would be impossible for deFuse to identify those events. From this list we selected 14 MapSplice predictions and eight FusionSeq predictions that we considered to have the highest likelihood of successful validation according to a variety of conservative criteria (Supplementary Methods, Text S1). For MapSplice, we attempted PCR validation for seven predictions from 50mer libraries CCC15, CCC16 and EMD6, and seven predictions from 75mer libraries SCH1, EMD5 and GRC5, with all 14 failing to produce a PCR product. For FusionSeq, we attempted PCR validation for eight predictions from CCC15, CCC16 and EMD6, three of which validated (Table 3).

**Table 3:** Fusions predictions compared between deFuse and FusionSeq.

library	5′ gene	3′ gene	FusionSeq	deFuse thresholds	deFuse classifier	PCR validated
CCC15	*UBR4*	*JMJD2B*	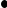	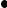	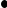	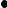
CCC15	*TMEM63A*	*NRD1*	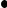	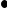	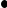	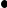
CCC15	*PVRL2*	*LMNA*	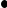	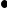	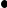	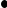
CCC15	*ARSB*	*DMGDH*	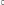	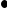	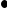	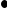
CCC15	*NUMB*	*ALDH6A1*	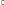	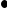	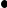	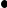
CCC15	*KIFC3*	*CNGB1*	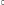	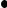	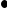	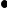
CCC15	*AFF4*	*LAMC3*	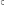	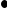	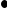	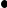
CCC16	*PAPOLA*	*HIP1R*	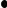	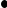	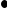	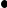
CCC16	*HPS5*	*APOO*	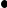	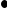	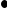	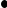
CCC16	*PPL*	*RBKS*	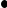	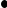	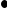	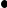
EMD6	*BCAS3*	*ARHGAP15*	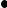	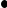	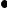	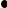
EMD6	*EIF4G3*	*LRRC8D*	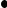	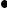	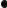	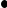
EMD6	*ROCK1*	*CMKLR1*	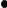	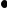	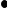	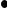
EMD6	*POLR2J2*	*CLMN*	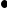	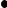	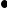	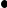
EMD6	*CAMK2G*	*DDX1*	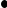	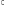	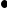	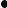
EMD6	*CYB5D2*	*ANKFY1*	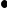	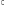	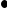	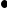
CCC15	*SLC38A10*	*ZCCHC11*	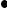	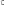	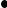	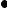
EMD6	*S100PBP*	*CAMK2G*	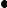	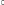	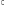	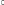
CCC15	*FARSA*	*RAD23A*	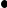	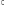	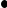	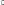
CCC16	*ITCH*	*DYNLRB1*	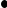	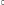	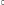	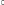
CCC16	*PIK3C2B*	*SMG5*	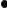	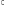	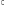	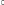

Comparison of deFuse using heuristic filters (deFuse thresholds) and deFuse using a classifier (deFuse classifier) with FusionSeq.

None of the 14 MapSplice predictions had corresponding deFuse predictions, filtered or unfiltered (blat with 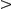
90% sequence identity). However seven of the eight FusionSeq predictions had a corresponding deFuse prediction, though all seven were filtered by the heuristic filters (Table 3). We initially trained the adaboost classifier on training data that did not include the seven deFuse predictions corresponding to these seven fusions identified by FusionSeq. We then used the resulting adaboost model to classify the seven deFuse predictions. The model successfully classified the three validated fusions as real, and erroneously classified as real one of the four predictions that failed to validate (Table 3). Subsequent analysis included the seven FusionSeq predictions as part of the 121 predictions used as training data as described in Methods.

In total there were 21 predictions with PCR results (17 positive and 4 negative) in CCC15, CCC16 and EMD6 upon which a quantitative comparison between FusionSeq and deFuse could be made. We computed the sensitivity and specificity on this data for deFuse-Threshold, deFuse-Classifier and FusionSeq. The sensitivity and specificity values were 82.3% and 100% for deFuse-Threshold; 100% and 94.4% for deFuse-Classifier; and 76.5% and 76.5% for FusionSeq (see Table 4).

**Table 4:** Comparison of accuracy metrics for FusionSeq and deFuse.

Method	P	N	TP	TN	FP	FN	Sens	Spec
deFuse-Threshold	17	4	14	4	0	3	82.3	100
deFuse-Classifier	17	4	17	3	1	0	100	94.4
FusionSeq	17	4	13	0	4	4	76.5	76.5

Comparison of accuracy between deFuse and FusionSeq on a subset of events predicted by either method in CCC15, CCC16 and EMD6. There were 21 PCR validations attempted including 17 positives (P) and 4 negatives (N). TP: true positives, TN: true negatives, FP: false positives, FN: false negatives, Sens = 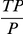, Spec = 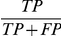.

#### Assessing the advantages of considering ambiguously aligning reads and avoiding reliance on known exon boundaries

We sought to establish the benefit of the maximum parsimony approach for resolving ambiguously aligning reads, and the dynamic programming based approach for aligning split reads to discover fusion boundaries. Each predicted fusion splice was annotated as coincident or not coincident with known ensembl exon boundaries. The fusion splices for eight of the 45 PCR validated fusions were not predicted to coincide with ensembl exon boundaries (Table 1), including *CMKLR1*-*HNF1A* and *CRADD*-*ERBB3* involving cancer associated *HNF1A* and *ERBB3* [24]–[26]. None of these eight gene fusions would be discoverable using a method that relied on the identification of reads split at known exon boundaries.

For each PCR validated fusion, we also calculated the number of spanning reads that align to a unique location in the genome, and considered the effect of an analysis restricted to considering only these reads. Such a theoretical analysis would have resulted in four fusions having lower than the threshold of five spanning reads (Table 1), and one of those fusions having no spanning reads. The *SMARCB1*-*WASF2* fusion involving *SMARCB1*, a gene known to be affected by genomic rearrangements in other cancers [27], would have only two reads in such an analysis. The *TYW1*-*HGSNAT* fusion with 41 spanning reads, ranked 10th by spanning read count out of the 45 PCR validate fusions, would have only three spanning reads in the restricted analysis.

An analysis that considered only uniquely aligning spanning reads and considered fusion splices at known exon boundaries would theoretically result in fewer false positives, as is apparent from the high validation rate in previous studies [10], [16]. However, such an analysis would be guaranteed to miss fusions in our datasets, including fusions involving previously described fusion partners. Considering ambiguously aligned spanning reads and performing an unbiased search for fusion splices can help to recover these false negatives, while more sophisticated techniques such as corroborating spanning and split read evidence can help to reduce the false positive rate without increasing the false negative rate.

### Rediscovery of known gene fusions

We evaluated the ability of deFuse to rediscover known gene fusions in publicly available RNA-Seq data. Using deFuse, we searched for the *TMPRSS2*-*ERG* fusion in the NCI-H660 prostate cell line dataset, the three fusions previously identified in the CML dataset (SRA accession: SRR018269) and the 11 fusions in melanoma libraries identified by Berger et al. [16]. Since seven of the fusions in the melanoma datasets are supported by fewer than five spanning reads, we altered the configuration of deFuse for the melanoma libraries such that only two spanning reads and one split read were required for deFuse to attempt assembly of a fusion boundary sequence. For all 15 fusions, deFuse was able to assemble the correct fusion boundary sequence. We evaluated the performance of deFuse using heuristic filters (deFuse-Thresholds), and deFuse using the adaboost classifier (deFuse-Classifier), when applied to the prostate, CML and melanoma datasets (Table 5). Since the training set for deFuse-Classifier includes the prostate, CML and melanoma fusions, we used a leave one out method classify each fusion.

**Table 5:** deFuse predictions for existing datasets with known fusions.

library	5′ gene	3′ gene	span count	split count	corrob. p-value	Split pos. p-value	split anchor p-value	deFuse correct sequence	deFuse thresholds	deFuse classifier probability
NCIH660	TMPRSS2	ERG	19	10	0.31	0.31	0.45	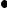	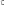	0.48
SRR018259	KCTD2	ARHGEF12	4	1	0.33	0.97	0.91	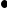	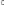	0.98
SRR018260	ITM2B	RB1	19	2	0.37	0.51	0.68	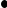	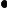	0.98
SRR018260	ANKHD1	C5orf32	2	2	0.45	0.09	0.05	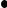	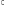	0.00
SRR018261	GCN1L1	PLA2G1B	4	1	0.35	0.57	0.62	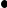	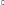	1.00
SRR018265	WDR72	SCAMP2	3	2	0.00	0.97	0.25	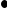	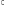	0.59
SRR018266	C1orf61	CCT3	54	17	0.12	0.56	0.56	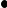	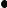	0.79
SRR018266	MIXL1	PARP1	2	1	0.18	0.25	0.19	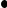	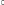	0.03
SRR018266	C11orf67	SLC12A7	43	24	0.92	0.55	0.75	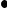	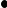	0.99
SRR018266	GNA12	SHANK2	29	9	0.58	0.24	0.34	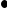	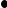	0.83
SRR018267	TLN1	C9orf127	3	1	0.08	0.71	0.76	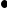	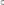	0.91
SRR018267	ALX3	RECK	4	6	0.72	0.25	0.45	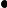	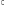	0.99
SRR018269	ABL1	BCR	91	14	0.68	0.68	0.67	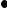	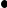	0.97
SRR018269	SLC44A4	BAT3	27	6	0.44	0.74	0.67	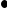	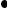	0.99
SRR018269	NUP214	XKR3	67	15	0.90	0.91	0.28	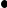	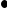	1.00

Results for an analysis of existing datasets using deFuse thresholds and deFuse classifier.

deFuse-Thresholds identifies 7 of the 15 known fusions, whereas deFuse-Classifier identifies 10 of the 15 fusions. Notably, *TMPRSS2*-*ERG* is not included in the deFuse-thresholds or the deFuse-Classifier results, primarily because the *TMPRSS2*-*ERG* prediction is an outlier on both the *Spanning read coverage* feature and the *Fusion boundary di-nucleotide entropy* feature. deFuse-Classifier assigns a probability of 0.48 to the *TMPRSS2*-*ERG* prediction. A probability threshold of 0.48 (instead of 0.81 as calculated in “Classification of ovarian and sarcoma predictions”) would result in a true positive rate of 93%, and false positive rate of 14%. These numbers suggest that our initially selected probability threshold of 0.81 may have been overly conservative given that we could have increased our true positive rate by 11% at the expense of only a 4% increase in false positive rate. Using a threshold of 0.48, deFuse-Classifier would recover 13 of the 15 events including *TMPRSS2*-*ERG*.

### Fusion boundaries coincident with interrupted expression show dominant expression of the fused gene

We sought to understand each fusion's impact on the expression patterns of the fused genes. For a given fusion boundary 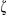, let 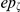 be the expression of exons on the preserved side of 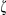, normalized by the length of those exons. Also let 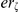 be the length normalized expression of the remaining exons, not predicted to be part of the fusion gene. We define the interrupted expression index 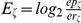 as the ratio of the expression of preserved versus remaining exons, analogous to the splicing index [28]. For each PCR-validated fusion boundary 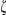 predicted for an ovarian dataset we calculated 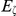 for all ovarian datasets and compared 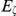 for the dataset with the predicted fusion to 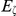 for the datasets without the fusion using a Wilcoxon test [28], resulting in 22 fusion events with at least one partner predicted as interrupted (p-values 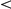
0.05, see Table 2 and Table S2).

Promoter exchanges are characterized by overexpression of the 3′ exons of a gene resulting from the replacement of 5′ regulatory regions [1]. For each PCR validated fusion we calculated whether the 3′ partner was expressed significantly higher in the dataset harbouring the fusion compared to other ovarian datasets (p-values 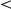
0.1, see Table S2, Table S6 and Table S7). We then overlapped the overexpression results with the interrupted expression results to find seven fusions representing potential promoter exchanges (Table 2). The remaining 15 expression-interrupting fusions represent either biallelic inactivations (for example, *HNF1A* described below) or dominant expression of the fusion allele (for example, *RREB1*-*TFE3* described below).

**Figure 6:**
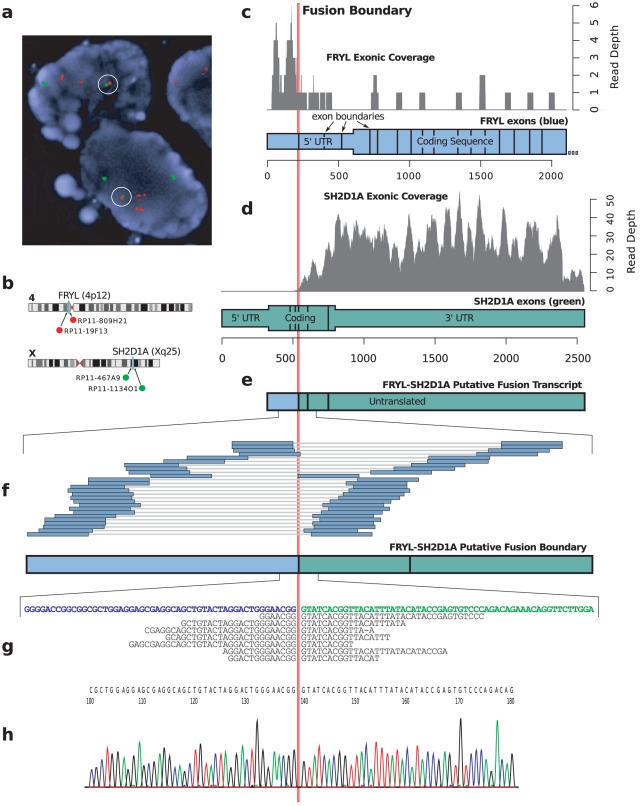
Evidence for the *FRYL*-*SH2D1A* fusion showing the validated fusion boundary (vertical red line). **a**) Validation evidence using a FISH come together assay, with fusion probes circled in white. **b**) FISH probe selection. **c**) *FRYL* exonic coverage showing fewer reads aligning after the fusion boundary. *FRYL* exons in blue with narrower boxes denoting untranslated sequence. **d**) *SH2D1A* exonic coverage showing significant coverage after the fusion boundary. *SH2D1A* exons in green with narrower boxes denoting untranslated sequence. **e**, *FRYL*-*SH2D1A* exons in blue or green depending on their origin, with the whole transcript predicted as untranslated. **f**) Positions of spanning reads supporting the fusion. **g**, Split alignments supporting the fusion prediction. **h**) Chromatogram of a sequenced PCR product supporting the fusion.

We sought to rule out genomic amplification as a mechanism of overexpression for the seven putative promoter exchanges. Analysis of Affy SNP6.0 genome data indicates that two of the 3′ partners, *SH2D1A* and *UMOD*, are in regions of genomic amplification (Table S3). Given that *UMOD* is not expressed in any other ovarian library (Table S9), a genomic amplification alone cannot explain *UMOD* expression in HGS4. For the *FRYL*-*SH2D1A* fusion in HGS3, a marked coincidence between the fusion boundary and an expression changepoint implies that only the fused copy of *SH2D1A* is expressed (Figure 6). FISH evidence for *FRYL*-*SH2D1A* indicates that at most one copy of the *FRYL*-*SH2D1A* fusion exists in the genome of each tumor cell (Figure 6), suggesting that amplification of the *SH2D1A* region is not the underlying cause of *SH2D1A* overexpression. *FRYL* expression is on average 670 fold higher than *SH2D1A* expression in the non-HGS3 ovarian libraries (Table S6), implying that the *FRYL* promoter would overexpress *SH2D1A*, were it fused to *SH2D1A*. In HGS3, *SH2D1A* expression is on average 36 fold higher than in other ovarian libraries, supporting the theory that the *FRYL* promoter is driving *SH2D1A* expression. The *FRYL*-*SH2D1A* fusion does not preserve the open reading frame of *SH2D1A*. Investigation of the functional impact of *FRYL*-*SH2D1A* and the other six promoter exchanges is ongoing.

### Evidence of previously described rearrangements in sarcoma and ovarian carcinoma data

We sought to identify previously described rearrangements in our sarcoma and ovarian carcinoma data. Although generally considered a breast cancer rearrangement, amplification of *ERBB2* has also been shown to occur in mucinous ovarian tumors [29]. In our ovarian cases, one mucinous tumor, *MUC1*, harbours a fusion between *ERBB2* and adjacent *PERLD1* caused by an underlying genomic inversion. CNV analysis of Affy SNP6.0 genome data predicted *ERBB2* to be highly amplified in the genome of *MUC1* (Table S3), and *ERBB2* expression is approximately 10 fold higher in *MUC1* than in any other ovarian library (Table S6). Since amplification of *ERBB2* requires replication of *ERBB2* across the genome, a reasonable explanation for the *ERBB2*-*PERLD1* fusion is that it is a secondary effect of the process of *ERBB2* amplification.

**Figure 7:**
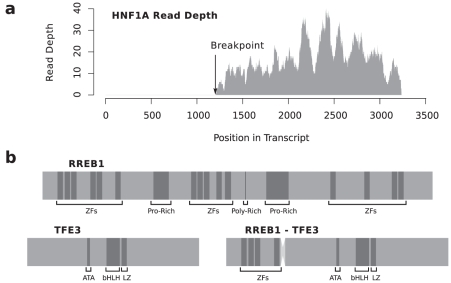
Fusions in sarcoma samples. **a**) Read depth across *HNF1A* exonic positions shows that only the region after the fusion boundary is being expressed, evidence of the possible biallelic inactivation of *HNF1A*. **b**) Putative *RREB1*-*TFE3* chimeric protein showing preservation of *TFE3's* basic helix-loop-helix (bHLH) leucine zipper (LZ) domain and N-terminal activation domain (ATA), in addition to 4 of *RREB1*'s zinc finger (ZF) motifs.

Analysis of the two epithelioid sarcomas and one intermediate grade myofibroblastic sarcoma produced five fusion predictions between non-adjacent genes, three involving genes previously described as translocated in cancer. The *CMKLR1*-*HNF1A* fusion is predicted to significantly interrupt expression of *HNF1A* (Table 2). In fact, there is no evidence of wild-type *HNF1A* expression in SARC1, indicating the possibility that the *CMKLR1*-*HNF1A* fusion transcript is evidence of a biallelic inactivation of *HNF1A* in SARC1 (Figure 7a). Biallelic inactivation of *HNF1A* has been previously reported to lead to aberrant activation of signalling pathways involved in tumorigenesis in human hepatocellular adenomas [26].

The *RREB1*-*TFE3* gene fusion found in the intermediate grade myofibroblastic sarcoma SARC3 fuses the first eight exons of *RREB1* to the last nine exons of *TFE3*, preserving the open reading frame of both *RREB1* and *TFE3*. The fusion is predicted to interrupt expression of *RREB1* (Table 2), indicating that *RREB1*-*TFE3* is the dominantly expressed *RREB1* allele. The underlying translocation leaves intact the DNA binding domain and N-terminal activation domain of *TFE3* (Figure 7b). *TFE3* is a known fusion partner in papillary renal cell carcinoma [30] and alveolar soft part sarcoma [31].

Finally, the *SMARCB1*-*WASF2* gene fusion found in SARC2 is predicted to produce a transcript that preserves the reading frame of both *SMARCB1* and *WASF2*. The predicted fusion protein would be composed of amino acids 1–209 of *SMARCB1*, which would preserve a DNA binding domain at 106–183 but interrupt a MYC binding domain at 186–245 [32], suggesting that the *SMARCB1*-*WASF2* fusion protein would retain only partial *SMARCB1* function. *SMARCB1* has been shown to be frequently inactivated in epithelioid sarcomas [27].

## Discussion

We have developed a new algorithmic method called deFuse for gene fusion discovery in RNA-Seq data. We evaluated deFuse on 40 ovarian cancer patient samples, one ovarian cancer cell line and three sarcoma patient samples. Using these data, we demonstrate with RT-PCR validated fusions how deFuse exhibits substantially better accuracy than two competing methods and that deFuse is able to discover gene fusions that are not discoverable by more simplistic methods. deFuse computes a set of 11 quantitative features used to characterize its predicted fusions. In our initial analysis we used heuristic, intuitively chosen thresholds to eliminate false positives and nominated expected true positives and false positive predictions for RT-PCR validation. This yielded a set of benchmark fusion predictions: 60 true positives and 61 true negatives that we in turn leveraged to train an adaboost classifier to more robustly and objectively identify real gene fusions from the features. The classifier yielded an AUC accuracy of 0.91. Importantly, the validated fusions in ovarian cancer represent the first reported gene fusions in that tumor type.

### Limitations

The lack of a sufficient number of positive and negative controls for a particular type of event, such as gene fusions, represents a major challenge when evaluating novel algorithms designed for discovery of those events. This challenge is exacerbated when the prediction set contains a much larger proportion of negatives than positives. We attempted to select candidates to enrich for positive examples to provide a balanced set of ground truth events with which to train our classifier. While this has inherent biases, only one in 40 randomly chosen predictions validated indicating that a completely unbiased selection would have yielded too few positives to robustly fit a classifier. We attempted to mitigate the acknowledged biases by using other software to find additional positives and also included the very limited set of published examples from the literature.

The main limitation of deFuse is the requirement of at least five discordant read pairs to nominate a gene fusion to the adaboost classifier. This will certainly miss fusions that have very low expression and may result in insensitivity to fusions from RNA-Seq datasets with minimal sequence generation. This is suggested by the results in “Rediscovery of known gene fusions”. However, sequencing platforms are increasing throughput at exponential rates and it will soon be rare for an RNA-Seq library to under-sample a transcriptome. Another potential limitation of deFuse is its reliance on an annotated set of genes. As such, it will not be able to discover fusions that involve loci that are not annotated as genes. Finally, deFuse relies on alignment to a reference as its primary analytical step. Thus deFuse would miss gene fusions involving completely novel sequences that may exist in a transcriptome library but are not represented in the reference used by the aligner. In such situations, de novo assembly based methods such as Trans-ABySS [33] may outperform deFuse.

### Conclusion

Full characterization of the mutational composition of cancer genomes will provide the opportunity to discover drivers of oncogenesis and will aid the development of biomarkers and drug targets for targeted therapy. As production of RNA-Seq data derived from tumor transcriptomes becomes routine, sophisticated techniques such as those used by deFuse will be required to identify the gene fusions that are part of each tumor's mutational landscape. As a first step in this process, we have identified gene fusions as a new class of features of the mutational landscape of ovarian tumor transcriptomes, in addition to discovering novel gene fusions in three sarcoma tumors.

## Supporting Information

Dataset S1 RT-PCR sequence traces.
(5.00 MB GZ)

Dataset S2 FISH images.
(4.55 MB GZ)

Dataset S3 MapSplice output.
(10.16 MB GZ)

Dataset S4 FusionSeq output.
(0.28 MB GZ)

Table S1 Table of all gene fusion predictions.
(1.97 MB TXT)

Table S2 Table of predicted interrupted genes.
(0.01 MB TXT)

Table S3 Table of predicted CNVs.
(0.64 MB TXT)

Table S4 FISH probe selection table.
(0.00 MB TXT)

Table S5 Table of Validation Sets and RT-PCR primers.
(0.06 MB TXT)

Table S6 Ovarian gene expression table.
(0.25 MB TXT)

Table S7 Sarcoma gene expression table.
(0.00 MB TXT)

Table S8 Table of positive and negative controls.
(0.10 MB TXT)

Table S9 UMOD aligned read counts.
(0.00 MB TXT)

Table S10 Gene names and their ensembl ids.
(0.51 MB TXT)

Text S1 Supplementary methods and analysis.
(0.58 MB PDF)
