# Supplementary figures and images for: deFuse: An Algorithm for Gene Fusion Discovery in Tumor RNA-Seq Data
Source: PLoS Comput Biol. 2011 May 19;7(5):e1001138. doi: 10.1371/journal.pcbi.1001138 (PMC3098195; doi:10.1371/journal.pcbi.1001138)

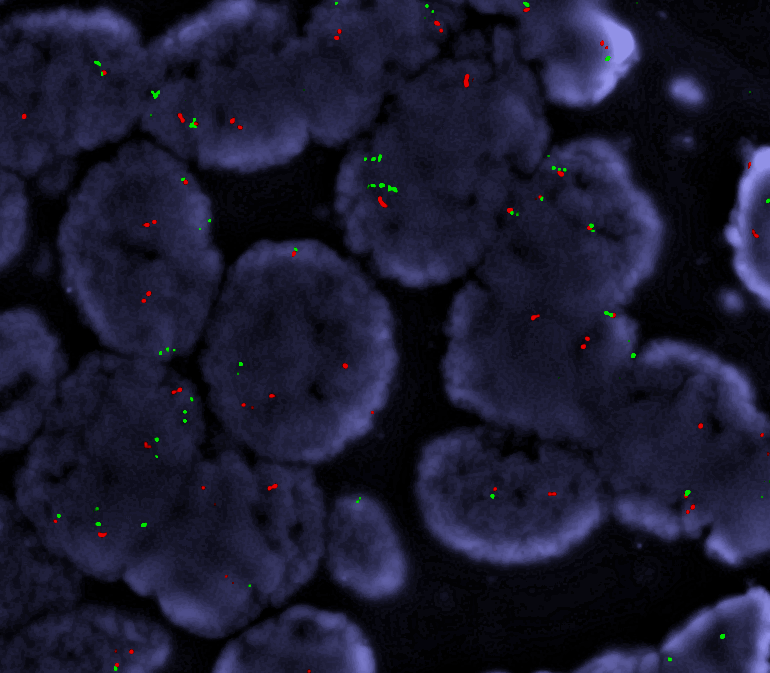

Supplement: Dataset S2 — FISH images. (4.55 MB GZ) [file pcbi.1001138.s002.gz › SupplementaryDataS9/BCAS3-ARHGAP15.001.A.tif]

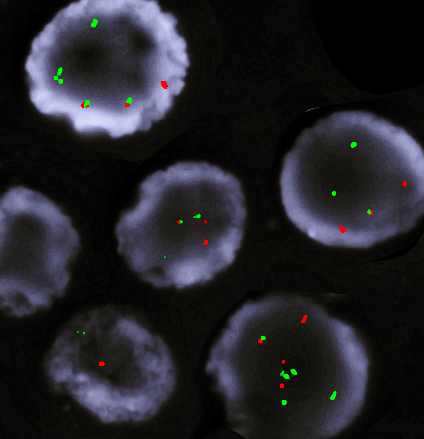

Supplement: Dataset S2 — FISH images. (4.55 MB GZ) [file pcbi.1001138.s002.gz › SupplementaryDataS9/c1orf61-MTHFD1.001.A.tif]

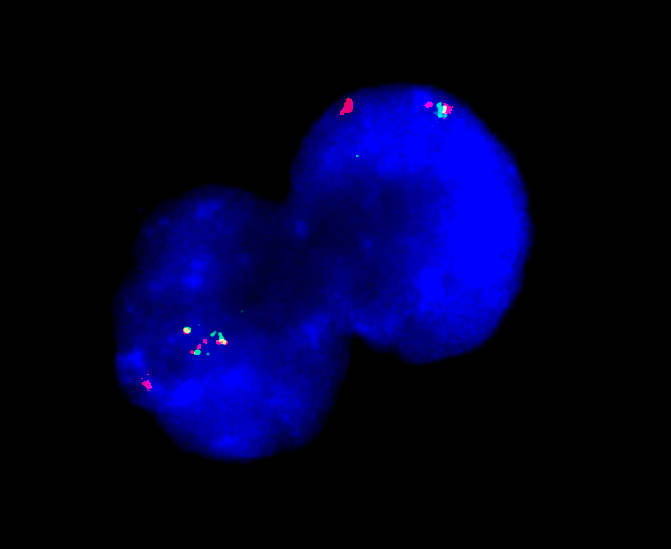

Supplement: Dataset S2 — FISH images. (4.55 MB GZ) [file pcbi.1001138.s002.gz › SupplementaryDataS9/CRADD-ERBB3.008.A.tif]

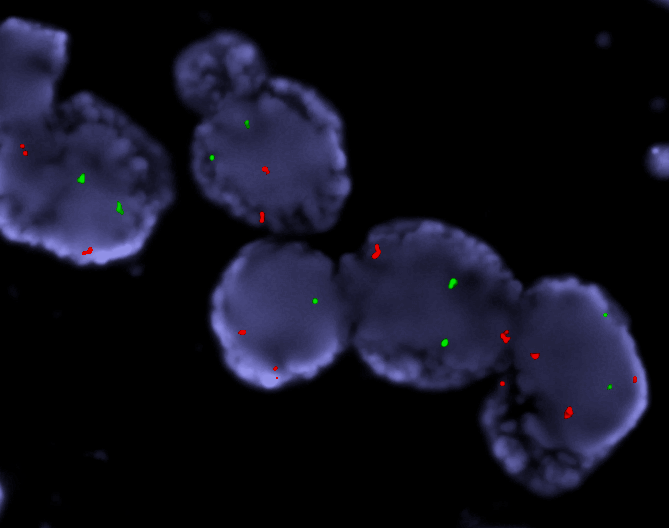

Supplement: Dataset S2 — FISH images. (4.55 MB GZ) [file pcbi.1001138.s002.gz › SupplementaryDataS9/DLEC1-EPCAM.004.A.tif]

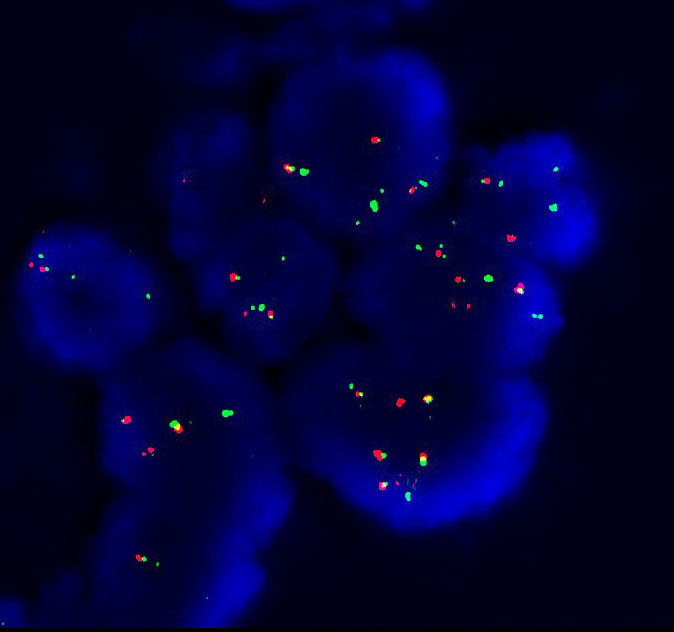

Supplement: Dataset S2 — FISH images. (4.55 MB GZ) [file pcbi.1001138.s002.gz › SupplementaryDataS9/FLNB-VPS8.tif]

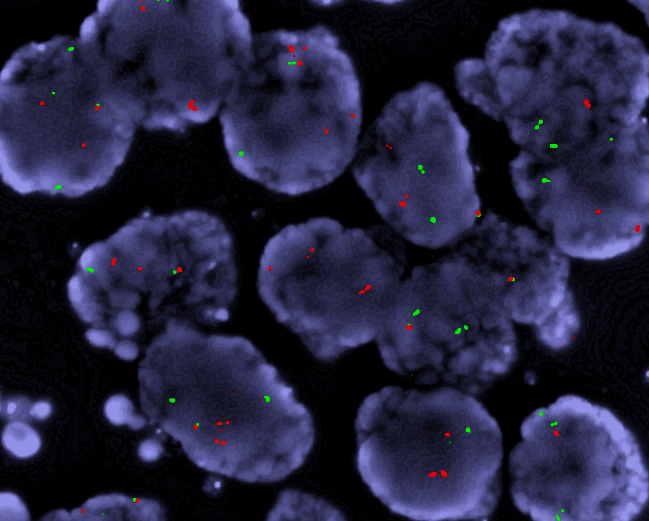

Supplement: Dataset S2 — FISH images. (4.55 MB GZ) [file pcbi.1001138.s002.gz › SupplementaryDataS9/FRYL-SH2D1A.001.A.tif]

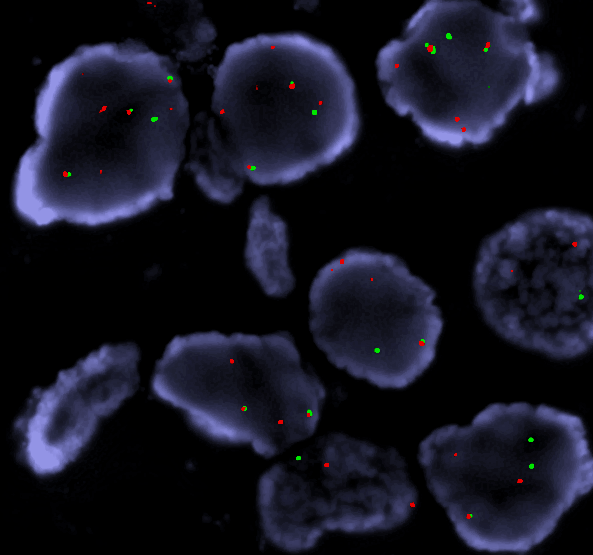

Supplement: Dataset S2 — FISH images. (4.55 MB GZ) [file pcbi.1001138.s002.gz › SupplementaryDataS9/H5S5-APOO.002.A.tif]

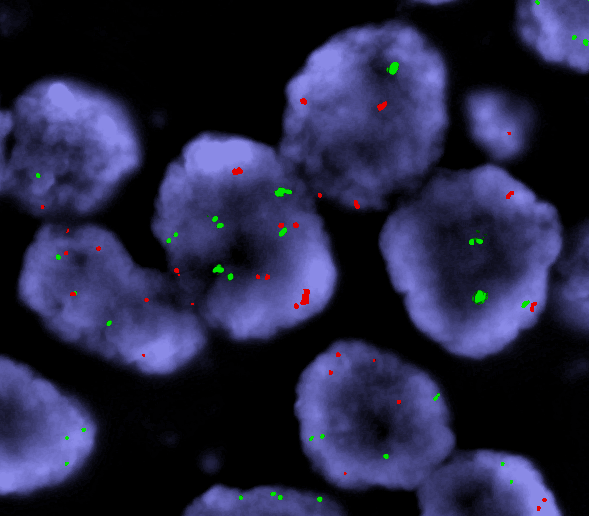

Supplement: Dataset S2 — FISH images. (4.55 MB GZ) [file pcbi.1001138.s002.gz › SupplementaryDataS9/HGSNAT-TYW1.008.A.tif]

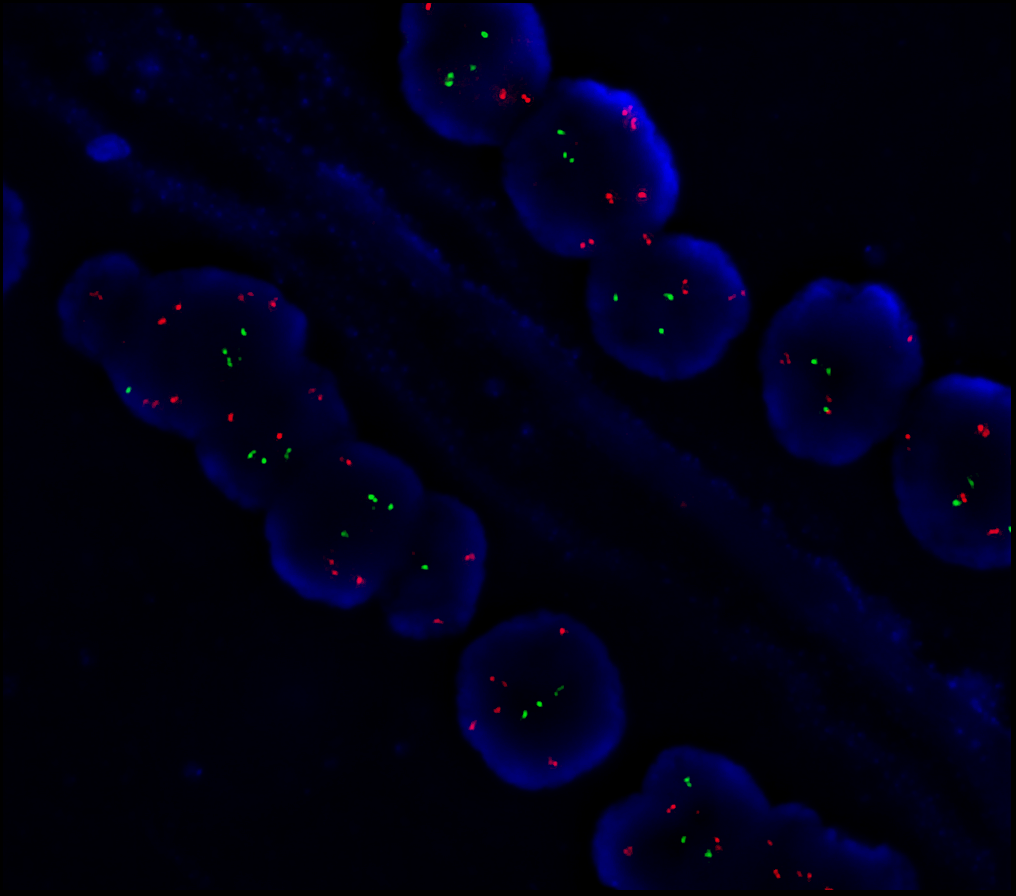

Supplement: Dataset S2 — FISH images. (4.55 MB GZ) [file pcbi.1001138.s002.gz › SupplementaryDataS9/LMNA-PVRL2.015.A.tif]

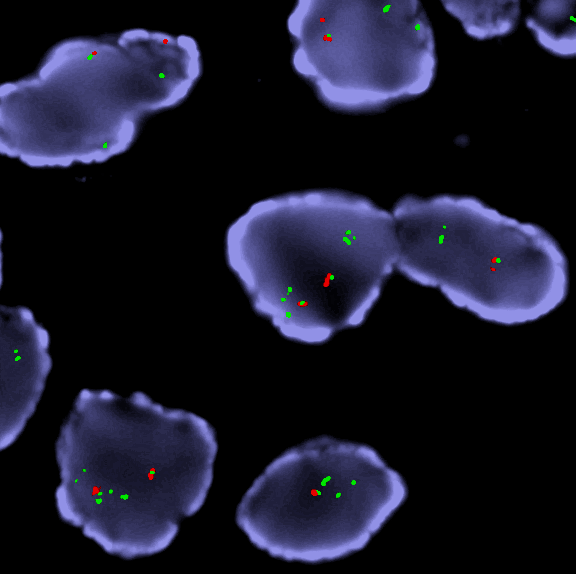

Supplement: Dataset S2 — FISH images. (4.55 MB GZ) [file pcbi.1001138.s002.gz › SupplementaryDataS9/PAPOLA-HIP1R.004.A.tif]

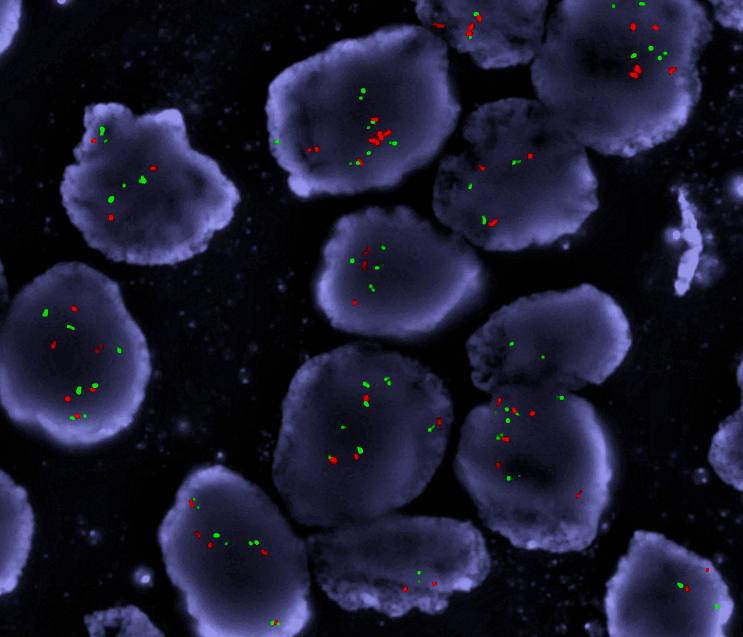

Supplement: Dataset S2 — FISH images. (4.55 MB GZ) [file pcbi.1001138.s002.gz › SupplementaryDataS9/PMEPA1-RPN2.001.A.tif]

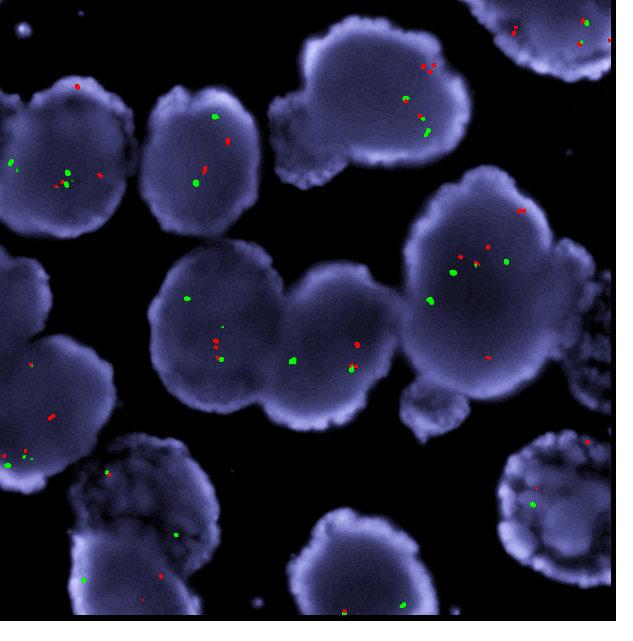

Supplement: Dataset S2 — FISH images. (4.55 MB GZ) [file pcbi.1001138.s002.gz › SupplementaryDataS9/PPL-RBKS.003.A.tif]

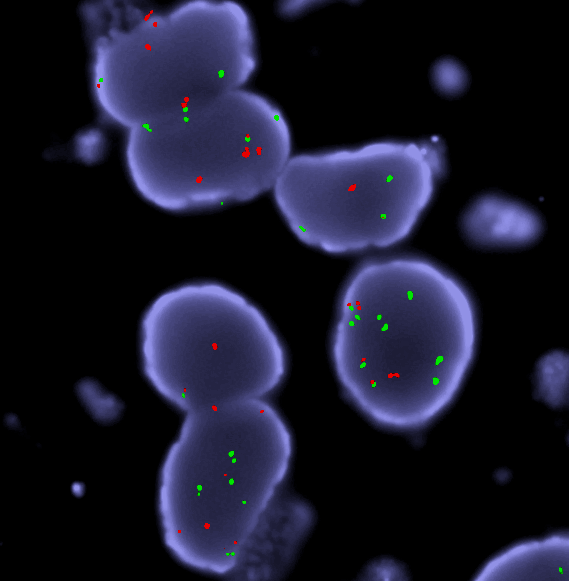

Supplement: Dataset S2 — FISH images. (4.55 MB GZ) [file pcbi.1001138.s002.gz › SupplementaryDataS9/RAB6A-USP43.002.A.tif]

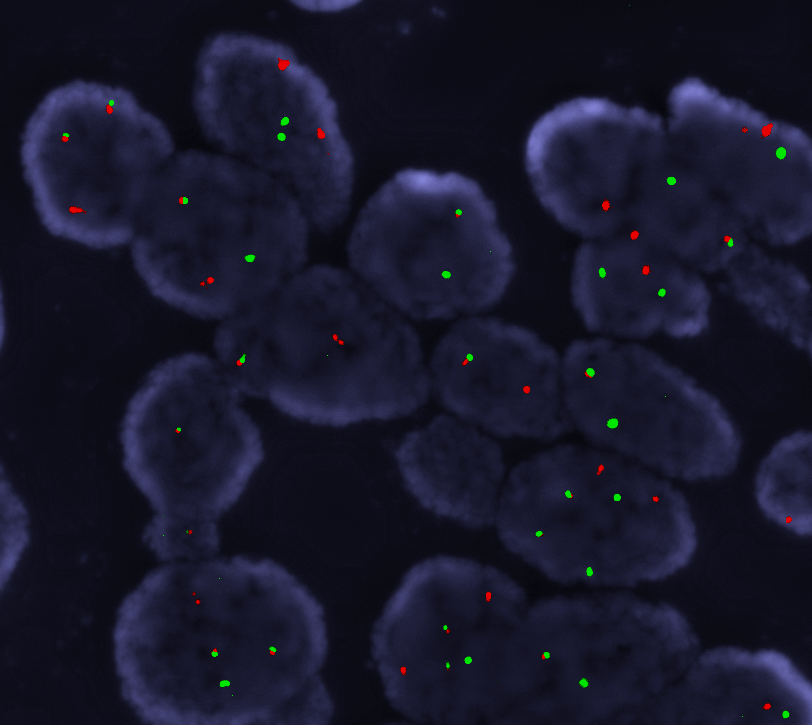

Supplement: Dataset S2 — FISH images. (4.55 MB GZ) [file pcbi.1001138.s002.gz › SupplementaryDataS9/ROCK1-CMKLR1.002.A.tif]

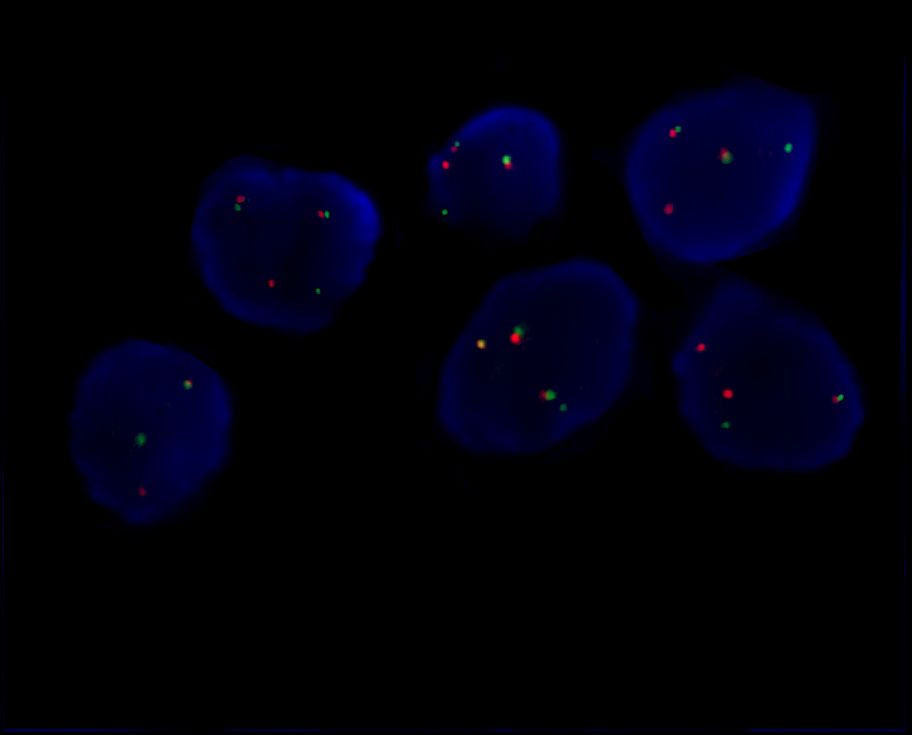

Supplement: Dataset S2 — FISH images. (4.55 MB GZ) [file pcbi.1001138.s002.gz › SupplementaryDataS9/RREB1-breakapart.jpg]

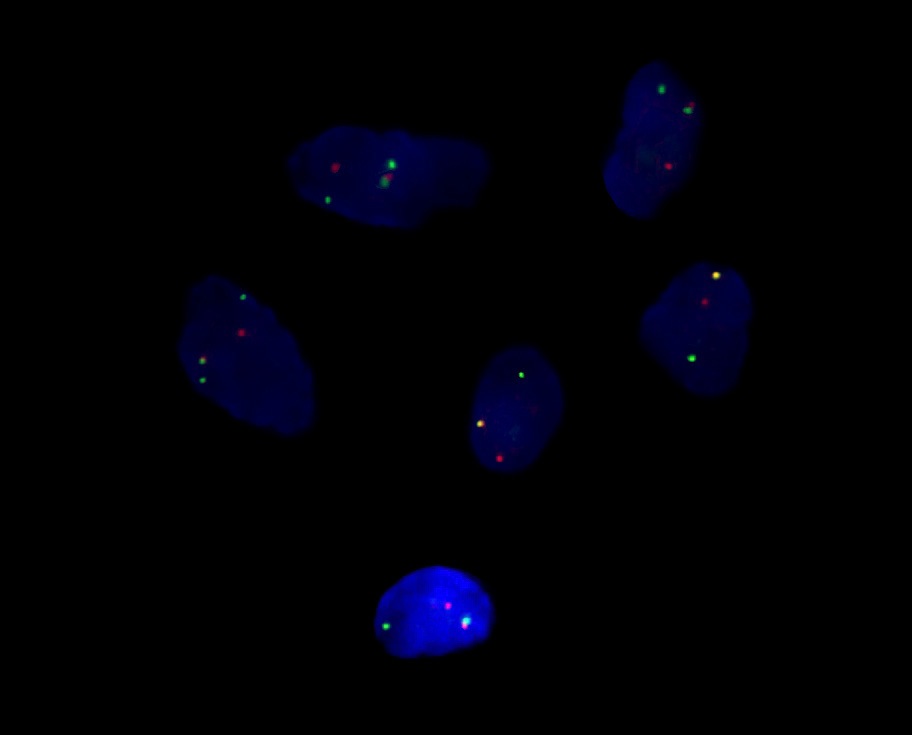

Supplement: Dataset S2 — FISH images. (4.55 MB GZ) [file pcbi.1001138.s002.gz › SupplementaryDataS9/TFE3-breakapart.jpg]

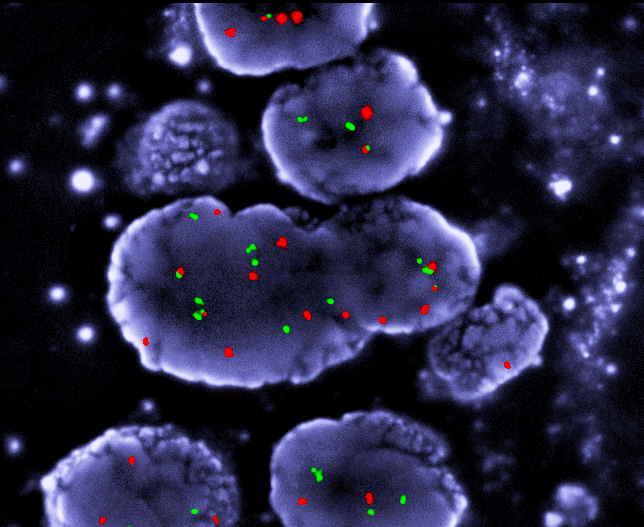

Supplement: Dataset S2 — FISH images. (4.55 MB GZ) [file pcbi.1001138.s002.gz › SupplementaryDataS9/TMEM63A-NRD1.002.A.tif]

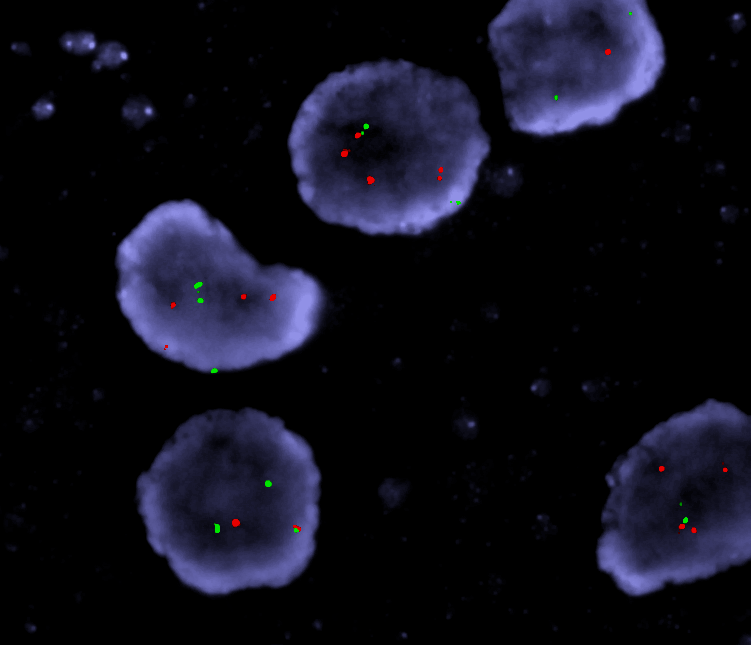

Supplement: Dataset S2 — FISH images. (4.55 MB GZ) [file pcbi.1001138.s002.gz › SupplementaryDataS9/UBR4-KDM4B.004.A.tif]
