# Supplementary material for: deFuse: An Algorithm for Gene Fusion Discovery in Tumor RNA-Seq Data
Source: PLoS Comput Biol. 2011 May 19;7(5):e1001138. doi: 10.1371/journal.pcbi.1001138 (PMC3098195; doi:10.1371/journal.pcbi.1001138)
Supplement: Text S1 — Supplementary methods and analysis. (0.58 MB PDF) [file pcbi.1001138.s015.pdf]

Supplementary Material for deFuse: an algorithm for gene fusion discovery in tumor RNA-Seq data

December 31, 2010

1 Glossary

Low Grade Serous (LGS) Ovarian cancer subtype characterized by small micropapillae that infiltrate ovarian stroma. Somatic KRAS, ERBB2, or BRAF mutations are found in two thirds of the cases and TP53 is rarely mutated.

High Grade Serous (HGS) Highly proliferative ovarian carcinoma subtype characterized by genomic instability due to TP53 loss and in some cases BRCA1/2 mutations. This cancer may originate in the fallopian tube.

Clear cell carcinoma (CCC) Ovarian carcinoma subtype characterized by large epithelial cells with abundant clear cytoplasm.

Endometrioid tumor (EMD) Ovarian carcinoma subtype composed of tubular glands bearing a close resemblance to benign or malignant endometrium.

Mucinous tumor (MUC) Ovarian carcinoma with similarities to mucinous colonic carcinomas.

Yolk sac tumor (YKS) Ovarian germ cell tumor that represents a proliferation of both yolk sac endoderm and extraembryonic mesenchyme.

Granulosa cell tumor (GRC) Ovarian tumors that arise from granulosa cells characterized by a single nucleotide variation in FOXL2.

Small cell hypercalemic (SCH) Ovarian cancer subtype characterized by diffuse sheets of cells punctured by variable numbers of follicle-like spaces. Often presents with hypercalcemia.

2 Summary of Supporting Information

Table S1 Table of all gene fusion predictions

Table S2 Table of predicted interrupted genes

Table S3 Table of predicted CNVs

Table S4 FISH probe selection table

Table S5 Table of Validation Sets and RT-PCR primers

Table S6 Ovarian gene expression table

Table S7 Sarcoma gene expression table

Table S8 Table of positive and negative controls

Table S9 UMOD aligned read counts

Table S10 Gene names and their ensembl ids

Dataset S1 RT-PCR sequence traces

Dataset S2 FISH images

Dataset S3 MapSplice Output

Dataset S4 FusionSeq Output

Text S1 This document

2.1 Table S1 - Table of all gene fusion predictions

The table of all gene fusion predictions is provided in tab delimited format with the following named columns labelled in the first line of the file. The order of the columns below does not correspond to the order in the file.

adjacent fusion between adjacent genes

break_adj_entropy_min minimum of break_adj_entropy1 and break_adj_entropy2

break_adj_entropy1 di-nucleotide entropy of the 40 nucleotide sequence adjacent to the fusion boundary in gene 1

break_adj_entropy2 di-nucleotide entropy of the 40 nucleotide sequence adjacent to the fusion boundary in gene 2

break_predict breakpoint prediction method, not currently used

breakpoint_homology number of homologous nucleotides at the fusion boundary

breakseqs_estislands_percident maximum percent identity of fusion sequence alignments to est islands

cdna_breakseqs_percident maximum percent identity of fusion sequence alignments to cdna

classification adaboost classifier result, TRUE for a real fusion, FALSE for an artifact

cluster_id random identifier assigned to each prediction

cnv_break1 a cnv breakpoint as determined using Affy SNP 6.0 genome arrays exists in gene 1 or within 2kb upstream or downstream

cnv_break2 a cnv breakpoint as determined using Affy SNP 6.0 genome arrays exists in gene 2 or within 2kb upstream or downstream

coding1 fusion splice / breakpoint in coding sequence of gene 1

coding2 fusion splice / breakpoint in coding sequence of gene 2

concordant_ratio proportion of spanning reads considered concordant by blat

deletion fusion produced by a genomic deletion

downstream1 fusion splice / breakpoint is downstream of gene 1

downstream2 fusion splice / breakpoint is downstream of gene 2

est_breakseqs_percident maximum percent identity of fusion sequence alignments to est

eversion fusion produced by a genomic eversion

exonboundaries fusion splice at exon boundaries

exonic1 fusion breakpoint in exonic sequence of gene 1

exonic2 fusion breakpoint in exonic sequence of gene 2

expression1 expression of gene 1 as number of concordant pairs aligned to exons

expression2 expression of gene 2 as number of concordant pairs aligned to exons

fish_validated fusion was validated by FISH

gene_align_strand1 alignment strand for spanning read alignments to gene 1

gene_align_strand2 alignment strand for spanning read alignments to gene 2

gene_chromosome1 chromosome of gene 1

gene_chromosome2 chromosome of gene 2

gene_end1 end position for gene 1

gene_end2 end position for gene 2

gene_name1 name of gene 1

gene_name2 name of gene 2

gene_start1 start of gene 1

gene_start2 start of gene 2

gene_strand1 strand of gene 1

gene_strand2 strand of gene 2

gene1 ensembl id of gene 1

gene2 ensembl id of gene 2

genome_breakseqs_percident maximum percent identity of fusion sequence alignments to genome

genomic_break_pos1 genomic position in gene 1 of fusion splice / breakpoint

genomic_break_pos2 genomic position in gene 2 of fusion splice / breakpoint

genomic_strand1 genomic strand in gene 1 of fusion splice / breakpoint, retained sequence upstream on this strand, breakpoint is downstream

genomic_strand2 genomic strand in gene 2 of fusion splice / breakpoint, retained sequence upstream on this strand, breakpoint is downstream

interchromosomal fusion produced by an interchromosomal translocation

interrupted_index1 ratio of coverage before and after the fusion splice / breakpoint in gene 1

interrupted_index2 ratio of coverage before and after the fusion splice / breakpoint in gene 2

interrupted1 fusion is predicted to interrupt expression of gene 1

interrupted2 fusion is predicted to interrupt expression of gene 2

intronic1 fusion splice / breakpoint is in an intronic region of gene 1

intronic2 fusion splice / breakpoint is in an intronic region of gene 2

inversion fusion produced by genomic inversion

library_name name of the RNA-Seq library

max_map_count maximum value for the number of alignment locations for each spanning read

mean_map_count mean value for the number of alignment locations for each spanning read

min_map_count minimum value for the number of alignment locations for each spanning read

num_multi_map number of spanning reads for which more than one alignment location exists

orf fusion combines genes in a way that preserves a reading frame

probability probability estimate produced by adaboost classifier that the gene fusion is real

read_through fusion involving adjacent potentially resulting from co-transcription rather than genome rearrangement

span_count number of spanning reads supporting the fusion

span_coverage_max maximum of span_coverage1 and span_coverage2

span_coverage_min minimum of span_coverage1 and span_coverage2

span_coverage1 coverage of spanning reads aligned to gene 1 as a proportion of expected coverage

span_coverage2 coverage of spanning reads aligned to gene 2 as a proportion of expected coverage

splicing_index1 number of concordant pairs in gene 1 spanning the fusion splice / breakpoint, divided by number of spanning reads supporting the fusion with gene 2

splicing_index2 number of concordant pairs in gene 2 spanning the fusion splice / breakpoint, divided by number of spanning reads supporting the fusion with gene 1

splitr_count number of split reads supporting the prediction

splitr_min_pvalue p-value, lower values are evidence the prediction is a false positive

splitr_pos_pvalue p-value, lower values are evidence the prediction is a false positive

splitr_sequence fusion sequence predicted by split reads

splitr_span_pvalue p-value, lower values are evidence the prediction is a false positive

upstream1 fusion splice / breakpoint is downstream of gene 1

upstream2 fusion splice / breakpoint is downstream of gene 2

utr3p1 fusion splice / breakpoint is in the 3 prime utr of gene 1

utr3p2 fusion splice / breakpoint is in the 3 prime utr of gene 2

utr3pexchange fusion is an exchange of 3 prime utrs

utr5p1 fusion splice / breakpoint is in the 5 prime utr of gene 1

utr5p2 fusion splice / breakpoint is in the 5 prime utr of gene 2

utr5pexchange fusion is an exchange of 5 prime utrs

validated validated by RT-PCR and sanger sequencing across the fusion boundary

2.2 Table S2 - Table of predicted interrupted genes

The table of all interrupted expression predictions for validated fusions is provided in tab delimited format with the following named columns labelled in the first line of the file.

library_name name of the RNA-Seq library

cluster_id identifier of the gene fusion prediction

genename name of the gene

gene ensembl id for the gene

before_size total length of exons before the fusion boundary and preserved in a putative fusion gene

after_size total length of exons after the fusion boundary and not preserved in a putative fusion gene

fusion_library_ratio interrupted expression index in library containing the fusion

other_ratios_mean mean interrupted expression indices in libraries not containing the fusion

other_ratios_stddev standard deviation of interrupted expression indices in libraries not containing the fusion

fusion_library_expr expression (reads per nucleotide) of the gene in library containing the fusion

other_expr_mean mean expression (reads per nucleotide) of the gene in libraries not containing the fusion

other_expr_stddev standard deviation of expression (reads per nucleotide) of the gene in libraries not containing the fusion

ratio_pvalue p-value associated with the Wilcoxon test that the interrupted expression index in the fusion library is higher than the interrupted expression indices in other libraries

expr_pval p-value associated with the Wilcoxon test that the expression of the gene in the fusion library is higher than the expression of the gene in other libraries

promotor_exchange ratio_pvalue < 0.05 and expr_pval < 0.1

2.3 Table S3 - Table of predicted CNVs

The table of predicted copy number variations (CNVs) is provided in tab delimited format with the following unnamed columns.

column 1 name of the RNA-Seq library

column 2 chromosome of the CNV

column 3 start position of the CNV

column 4 end position of the CNV

column 5 number of probes for the CNV

column 6 median log ratio copy number, <0 for loss >0 for gain

2.4 Table S4 - FISH probe selection table

The table of all FISH experiments and their result is provided in tab delimited format with the following named columns labelled in the first line of the file.

library_name name of the RNA-Seq library

gene1 gene 1 of the fusion

gene1_cyto_band cytogenetic band(s) for gene 1

gene1_bac1 BAC 1 for gene 1 (ucsc genome browser)

gene1_bac2 BAC 2 for gene 1

gene2 gene 2 of the fusion

gene2_cyto_band cytogenetic band(s) for gene 2

gene2_bac1 BAC 1 for gene 2

gene2_bac2 BAC 2 for gene 2

result result of the FISH validation

2.5 Table S5 - Table of Validation Sets and RT-PCR primers

The table of RT-PCR primers and validation results is provided in tab delimited format with the following named columns labelled in the first line of the file.

library_name name of the RNA-Seq library

gene1 gene 1 of the fusion

gene2 gene 2 of the fusion

gene1_id ensembl gene id for gene 1

gene2_id ensembl gene id for gene 2

defuse_id deFuse id if predicted by deFuse

mapsplice_id MapSplice id if MapSplice was run on this library, and MapSplice predicted this fusion

fusionseq_id FusionSeq id if FusionSeq was run on this library, and FusionSeq predicted this fusion

assembled_sequence assembled sequence as detailed in the main text for deFuse predictions, Section 4.11 for MapSplice predictions, or Section 4.10 for FusionSeq predictions

forward_primer forward primer used for RT-PCR

reverse_primer reverse primer used for RT-PCR

amplicon_size size of amplicon that should result from successful RT-PCR

comment additional information about the RT-PCR result, if necessary

final_result final result of RT-PCR and sequencing

validation_set validation set that this fusion belongs to, see main text

2.6 Table S6 - Ovarian gene expression table

The table of ovarian gene expression estimated from RNA-Seq is provided in tab delimited format. Genes are in the first column, and subsequent columns contain the expression estimate for that gene for each library.

2.7 Table S7 - Sarcoma gene expression table

The table of sarcoma gene expression estimated from RNA-Seq is provided in tab delimited format. Genes are in the first column, and subsequent columns contain the expression estimate for that gene for each library.

2.8 Table S8 - Table of positive and negative controls

The table of all positive and negative control deFuse predictions is provided in tab delimited format with named columns as for Table S1, with the following exceptions. The probability and classification columns are not included. A column named leave_one_out_probability is included corresponding to the probability estimate calculated for the given fusion when the adaboost model is trained on all but the given fusion and then used to classify that fusion.

2.9 Table S9 - UMOD aligned read counts

A table of read counts for each transcript for each library is provided in tab delimited format. The first column is the library name, the following 4 columns are the counts of number of reads aligning in paired end mode to the 4 transcript variants of *UMOD*.

2.10 Table S10 - Gene names and their ensembl ids

A table of ensembl gene identifiers and their corresponding gene names is provided in tab delimited format with the following named columns labelled in the first line of the file.

gene_name gene name

ensembl_id ensembl gene identifier

2.11 Dataset S1 - RT-PCR sequence traces

The sequence traces for all fusions successfully validated by RT-PCR are provided in the form of ab1 files.

2.12 Dataset S2 - FISH images

FISH images are provided for all attempted FISH experiments.

2.13 Dataset S3 - MapSplice Output

Two files are provided for each of the 6 libraries for which MapSplice was used to predict fusions. The fusion.junction file corresponds to the similarly named file produced by MapSplice. The junctions.txt file contains a list of all predicted splice sites calculated by parsing the CIGAR strings of each alignment in the alignments.sam file produced by MapSplice.

2.14 Dataset S4 - FusionSeq Output

The confidence.gfr file is provided for each of the 3 libraries for which FusionSeq was used to predict fusions.

3 Supplementary Results

3.1 Towards a classifier for gene fusions predictions

We sought to develop a classifier for gene fusion predictions so that we would not have to rely on arbitrary thresholds. We selected the following 11 features, described in detail in section 4.7. We chose to not select features that could be related to expression, such as the number of split or spanning reads, since we did not wish to bias the classifier towards highly expressed fusions.

- Spanning read coverage
- Split position p-value
- Minimum split anchor p-value

- Corroboration p-value
- Concordant ratio
- Fusion boundary di-nucleotide entropy
- Fusion boundary homology
- cDNA adjusted percent identity
- Genome adjusted percent identity
- EST adjusted percent identity
- EST islands adjusted percent identity

We established whether each feature could be used to discriminate between true and false positives by plotting histograms of each feature for the 121 predictions in the example dataset (figure 1).

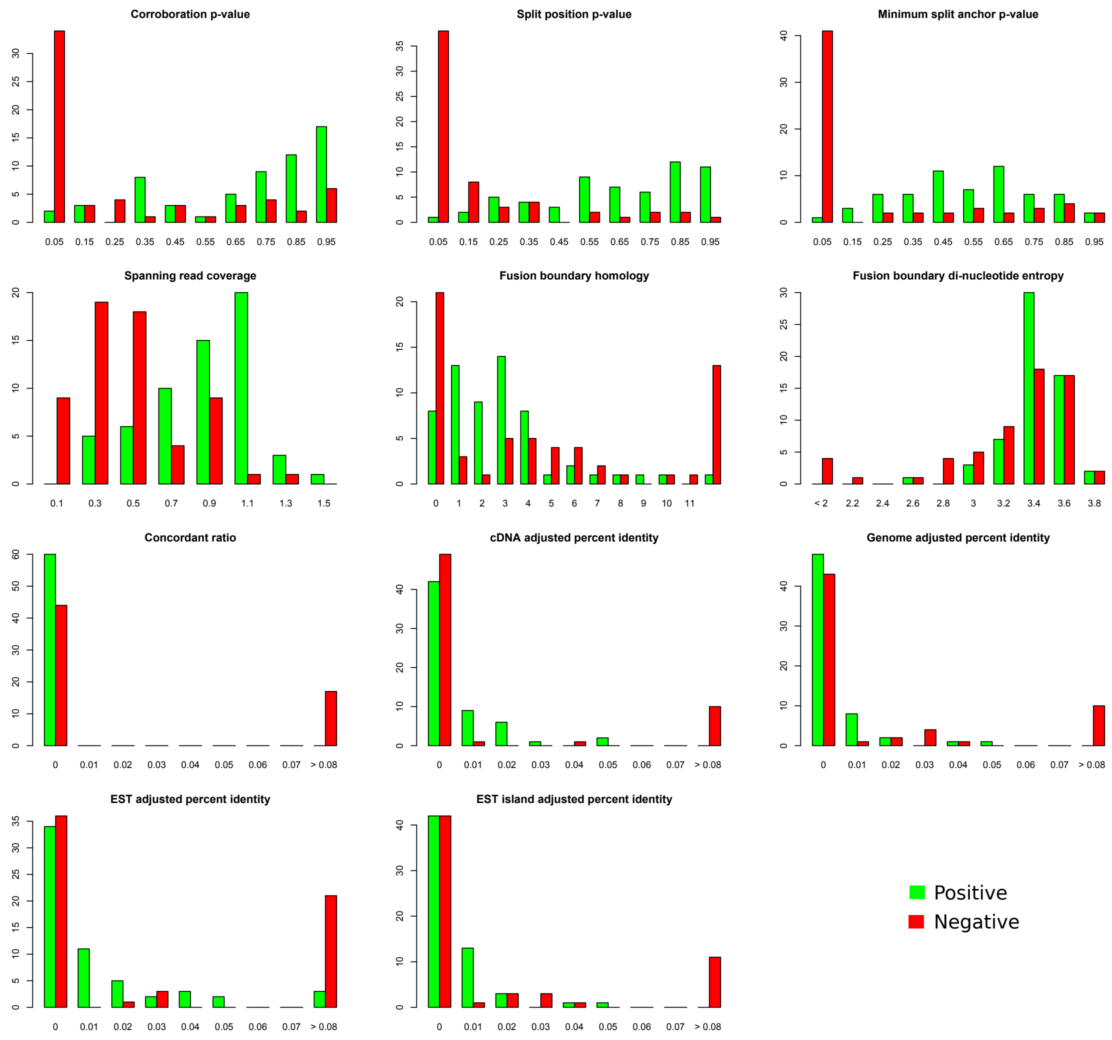

Figure 1: Histograms of each feature for all 121 predictions in the example dataset of 60 positive and 61 negative predictions

4 Supplementary Computational Methods

4.1 Conditions for considering discordant alignments to have originated from reads spanning the same fusion boundary

Let r be the read length, $fivep(a_X)$ be the aligned position in transcript X of the 5' end of the read and let $strand(a_X)$ be the strand of that alignment a_X. Then the fusion boundary region is given by equation 1.

$$br(a_X) = \begin{cases} [fivep(a_X) + r \ , \ fivep(a_X) + l_{max} - r] & \text{if } strand(a_X) = + \\ [fivep(a_X) - l_{max} + r \ , \ fivep(a_X) - r] & \text{if } strand(a_X) = - \end{cases} \quad (1)$$

Let a_X, a_Y, b_X and b_Y be the alignments to transcript X and Y of paired end reads a and b. We define the *overlapping boundary region condition* as the condition that the fusion boundary regions in each transcript must overlap in order to consider paired end reads a and b to have originated from the same fusion transcript. The overlapping boundary region condition ensures that there exists a valid location for the fusion boundary in transcript X and transcript Y that would simultaneously explain both paired end alignments. Included in the overlapping boundary region condition is the condition that $strand(a_X) = strand(a_Y)$ and $strand(b_X) = strand(b_Y)$. The overlapping boundary region condition is defined specifically as given in equation 2.

$$(br(a_X) \cap br(b_X) \neq \emptyset) \wedge (br(a_Y) \cap br(b_Y) \neq \emptyset) \quad (2)$$

Suppose now that transcripts X and Y are concatenated together as fusion transcript XY with a $+-$ alignment configuration (alignments are to the $+$ strand of X and the $-$ strand of Y). The location of the fusion boundary in each transcript is unknown, as is the variable d that corresponds to the distance between the two fusion boundaries in the concatenated sequence. The fragment lengths l_a and l_b of fragments a and b are unknown also. However, it is possible to calculate the difference between the fragment lengths as $|l_a - l_b| = |z_a - z_b|$

as shown in figure 2. We define the *similar fragment length condition* as the constraint that $|l_a - l_b|$ must be no more than $l_{max} - l_{min}$ for us to consider paired end reads a and b to have originated from the same fusion transcript.

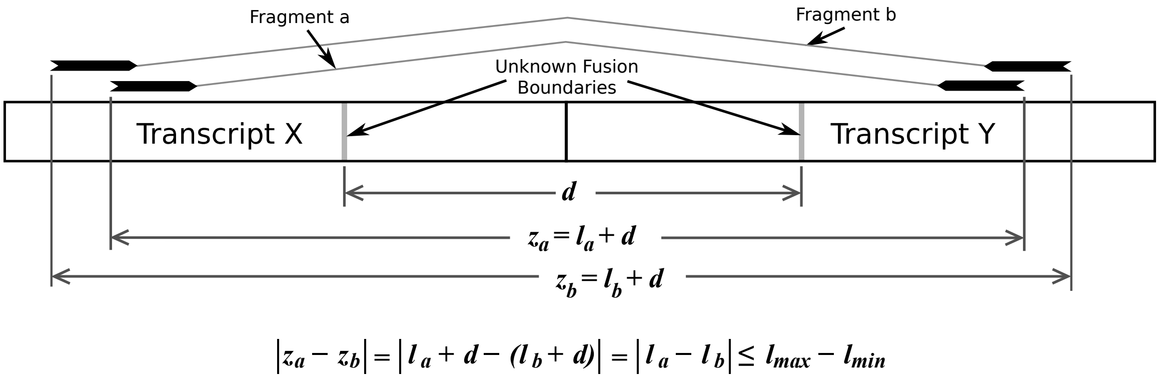

Figure 2: Fragment length difference can be calculated as $|z_a - z_b|$.

Trivially, if XY produces a $-+$ alignment configuration then YX will produce a $+-$ configuration and should be considered instead. However, it may also be interesting to consider the situation in which XY results in a $--$ or $++$ configuration because although the prediction may not represent a chimeric transcript with preserved open reading frame, it may represent an expressed structural variation or gene interruption. For this situation, a $+-$ configuration can be obtained by considering the reverse complement of either X or Y and recalculating the alignment positions to that reverse complemented sequence.

In practise, however, it is not necessary to remap the position of each alignment to the concatenated sequence described above, since any offset added to the positions of alignments to X or Y will be incorporated into the value d and will cancel out when calculating $|z_a - z_b|$. For the same reason, if it is necessary to reverse complement either X or Y, all that is required is to consider the negation of the positions of alignments to whichever of X or Y it was necessary to reverse complement, since any additional offset will be incorporated into the value d, and will cancel out. The value z_a (and z_b) can be calculated, with consideration for the strand of the alignments, using equation 3. Note that this formulation of the similar fragment length condition is equivalent to that given in the main text, and allows for easier calculation of maximal valid clusters using the method in 4.2.

$$z_a = \begin{cases} fivep(a_Y) + fivep(a_X) & \text{if } strand(a_X) = strand(a_Y) \\ fivep(a_Y) - fivep(a_X) & \text{if } strand(a_X) \neq strand(a_Y) \end{cases} \quad (3)$$

4.2 Generating Maximal Valid Clusters

We provide a polynomial time algorithm for calculating a set of clusters of paired end alignments, such that any two paired end alignments satisfy the overlapping boundary region and similar fragment length conditions, and such that those clusters are maximal.

Let $\mathcal{G}$ be the set of transcripts under consideration. Let $\mathcal{S} = \{+, -\}$ be the set of strands. Let $A_{X,Y,S,T}$ be the set of alignments such that one end finds at least one alignment to strand S of transcript X and the other end finds at least one alignment to strand T of transcript Y. Consider all distinct sets $A_{X,Y,S,T} \neq \emptyset$. Let A_X be the alignments to transcript X and A_Y be the alignments to transcript Y. Maximal paired end alignment clusters $P_{X,Y,S,T}$ satisfying both conditions can be computed in polynomial time as follows.

1. Create the fusion boundary region clusters C_X for transcript X. The fusion boundary region clusters can be created using a polynomial time algorithm as described in [2], reiterated here. Fusion boundary regions $br(A_X)$ are sorted by their start coordinate. Clustering proceeds by adding regions in left to right order to cluster C_X^k until a region is encountered that does not overlap with all other regions in C_X^k. Cluster C_X^k is kept unless it is a proper subset of C_X^{k-1}. Cluster C_X^{k+1} is initialized to $C_X^k \setminus a$ where a is the region in C_X^k with the leftmost end coordinate and the process repeats. Repeat for transcript Y creating C_Y.

2. Create clusters of paired end alignments D_{C_X,C_Y} where every paired end alignment $a \in D_{C_X,C_Y}$ satisfies $a \in C_X \wedge a \in C_Y$. For any D_{C_X,C_Y} it should be true that any two paired end alignments in D_{C_X,C_Y} satisfy the overlapping boundary region condition.

3. Refine clusters of paired end alignments D_{C_X,C_Y} into clusters of paired end alignments $\{D^i_{C_X,C_Y}\}$ that also satisfy the similar fragment length condition. For each paired end alignment a in D_{C_X,C_Y} calculate the value z_a. Sort the alignments by z and use a sliding window of size $l_{max} - l_{min}$ to calculate clusters $\{D^i_{C_X,C_Y}\}$. Specifically, proceed

by adding alignments to cluster $D^k_{C_X,C_Y}$ in order of increasing z while maintaining the property that the difference between the lowest and highest z values in $D^k_{C_X,C_Y}$ is less than or equal to $l_{max} - l_{min}$. Cluster $D^k_{C_X,C_Y}$ is kept unless it is a proper subset of $D^{k-1}_{C_X,C_Y}$. Cluster $D^{k+1}_{C_X,C_Y}$ is initialized to $D^k_{C_X,C_Y} \setminus a$ where a is the paired end alignment with the lowest z value.

4. Remove any cluster that is the subset of another cluster. Let $P_{X,Y,S,T} = \{D^i_{C_X,C_Y}\}$ be the resulting set of clusters. It can be easily verified that $P_{X,Y,S,T}$ is the set of maximal paired end alignment clusters satisfying both conditions.

4.3 Split read boundary sequence prediction

Let $C_{X,Y,S,T}$ be a paired end alignment cluster that is evidence between strand S of transcript X and strand T of transcript Y. Let A_X and A_Y be the end alignments of each paired end to transcripts X and Y respectively. Let $br(A_X) = \cap_{a_X \in A_X} br(a_X)$ and $br(A_Y) = \cap_{a_Y \in A_Y} br(a_Y)$. For each alignment with one end aligning to transcript X we calculate the *mate alignment region* denoted $mate(a_X)$ as in equation 4.

$$mate(a_X) = \begin{cases} \Big[fivep(a_X) + l_{min} - r \ , \ fivep(a_X) + l_{max}\Big] & \text{if } strand(a_X) = + \\ \\ \Big[fivep(a_X) - l_{max} \ , \ fivep(a_X) - l_{min} + r\Big] & \text{if } strand(a_X) = - \end{cases} \tag{4}$$

For each alignment with one end aligning to transcript X, if $br(A_X) \cap mate(a_X) \neq \emptyset$ then add the sequence of the end that does not align to transcript X to M_X. Repeat the process for transcript Y to create M_Y. Create the sequence S_X by extracting the sequence of transcript X in the range $br(A_X)$ expanded by r on each side.. Repeat for transcript Y to create S_Y. Reverse complement S_Y if $S = T$. Reverse complement the sequences in M_X. Reverse complement the sequences in M_Y if $S \neq T$. For each candidate split read $r \in M_X \cup M_Y = M$ align r to S_X using dynamic programming based local alignment and penalizing initial gaps in the end sequence. Repeat with the reverse of sequence r and the reverse of sequence S_Y (see supplementary section 4.4). Proceed as described in the main text of the paper.

4.4 Dynamic programming matrix definition

We use dynamic programming based local alignment penalizing initial gaps in the read sequence as part of the method for finding read sequences split by the fusion boundary. Let $\delta(p, q) = m$ if $p = q$ otherwise $\delta(p, q) = u$, thus m is the match score. Let g be the score given for a gap in either the read sequence of the transcript sequence. Let r be the read sequence and S the reference sequence on one side of the fusion boundary. The dynamic programming matrix may be defined as follows [8].

$$\begin{array}{ll} D(i,0) = 0 & 0 \le i \le |S| \\ D(0,j) = D(i,j-1) + g & 0 < j \le |r| \\ D(i,j) = \max \begin{cases} D(i-1,j-1) + \delta(p,q) \\ D(i-1,j) + g \\ D(i,j-1) + g \end{cases} & 0 < i \le |S|,\ 0 < j \le |r| \end{array} \tag{5}$$

4.5 Covariance between the lengths of fragments spanning a fusion boundary

We do not assume that the set of fragment lengths $\{l_i\}$ of paired end reads spanning the same fusion boundary are drawn *independently* from the fragmet length distribution $P(L)$. Thus the variance of $\bar{l}$ includes a covariance term $Cov(L_1, L_2)$ as given by equation 6. The covariance $Cov(L_1, L_2)$ represents the degree to which two fragments overlapping the same position are likely to have the same length.

$$Var(\bar{L}) \ = \ nVar(L) + \left(1 - \frac{1}{n}\right) Cov(L_1, L_2) \tag{6}$$

We estimate the covariance between the lengths of two fragments originating from the same location in the transcriptome using concordant alignments to cDNA. Concordant alignments to cDNA often contain paired end alignments that are consistently aligned to the wrong splice variant causing some alignments to imply the wrong fragment length. In an attempt

to mitigate this affect we only consider paired end alignments for which the implied fragment length is in the range $[\mu - 3\sigma \ \mu + 3\sigma]$ where μ and σ are the mean and standard deviation of inferred fragment length distribution. We begin by selecting n positions in the transcriptome at random. For each position we select at random, if they exist, two paired end alignments with one end aligning entirely to the left and one end aligning entirely to the right of that position. Let the fragment lengths implied by the two paired end alignments selected for position i be given by l_{i1} and l_{i2}. Equation 7 is used to estimate the covariance between the two random variables L_1 and L_2 representing the fragment lengths of two reads spanning the same fusion boundary.

$$\hat{Cov}[L_1, L_2] = \frac{\sum_i l_{i1} l_{i2}}{n} - \frac{\sum_i l_{i1} \sum_j l_{j2}}{n^2} \tag{7}$$

4.6 Covariance between split read statistics for reads split by a fusion boundary

We do not assume that the values p_i calculated for reads split by a fusion boundary are drawn *independently* from a uniform distribution. To model dependency we estimate the covariance $Cov(p_i, p_j)$. We begin by selecting n positions in the transcriptome at random. For each position we select at random, if they exist, two paired end alignments with one end overlapping that position by at least n_{anchor} nucleotides. We calculate p_1 and p_2 for both of these split alignments as given by equation 10. Equation 8 is then used to estimate the covariance between two random variables P_1 and P_2 representing p_i values of two reads split by the same fusion boundary. An equivilent analysis is used to estimate $\hat{Cov}(Q_1, Q_2)$ for q_i values as calculated by equation 10.

$$\hat{Cov}(P_1, P_2) = \frac{\sum_i p_{i1} p_{i2}}{n} - \frac{\sum_i p_{i1} \sum_j p_{j2}}{n^2} \tag{8}$$

4.7 Features

For each fusion prediction we calculate a number of features to assist in the discrimination between real fusions and false positives.

Spanning read count Number of reads spanning the fusion boundary.

Spanning read coverage Normalized spanning read coverage (section 4.7.1).

Split read count Number of reads split by the fusion boundary.

Split position p-value P-Value for the hypothesis that the *split position* statistic was calculated from split reads that are evenly distributed across the fusion boundary (section 4.7.2).

Minimum split anchor p-value P-Value for the hypothesis that the *minimum split anchor* statistic was calculated from split reads that are evenly distributed across the fusion boundary (section 4.7.2).

Corroboration p-value P-Value for the hypothesis that the lengths of reads spanning the fusion boundary were drawn from the fragment length distribution (section *Corroborating spanning and split read evidence* in the main text).

Concordant ratio Proportion of spanning reads supporting a fusion that have a concordant alignment using blat with default parameters.

Fusion boundary di-nucleotide entropy Di-nucldeotide entropy calculated 40 nt upstream and downstream of the fusion boundary for the predicted sequence, taking the minimum of both values (section 4.7.3) .

Fusion boundary homology Number of homologous nucleotides in each gene at the predicted fusion boundary (section 4.7.4).

cDNA adjusted percent identity Maximum adjusted percent identity (section 4.7.5) for the alignments of the predicted sequence to any cDNA.

Genome adjusted percent identity Maximum adjusted percent identity (section 4.7.5) for the alignments of the predicted sequence to the genome.

EST adjusted percent identity Maximum adjusted percent identity (section 4.7.5) for the alignments of the predicted sequence to any EST.

EST island adjusted percent identity Maximum adjusted percent identity (section 4.7.5) for the alignments of the predicted sequence to any EST island (section 4.7.6).

4.7.1 Normalized spanning read coverage

For each fusion partner gene X we calculate c_X, the number of nucleotides matched in X by at least one of the prediction's spanning reads alignments. We then normalize c_X by the expected coverage $l_{avg} - r_{min}$ where l_{avg} is the mean fragment length and r_{min} is the minimum read length. The *normalized spanning read coverage* for a prediction is the minimum of the normalized coverage calculated for each gene predicted as fused (equation 9). PCR duplicates of poor quality reads, or systematic alignment errors for small homologous regions are expected to result in smaller values for the normalized spanning read coverage than predictions representing real fusions.

$$\text{Normalized spanning read coverage} = \frac{\min(c_X, c_Y)}{l_{\text{avg}} - r_{\text{min}}} \tag{9}$$

4.7.2 Split position p-value and minimum split anchor p-value

Split read alignments are prone to systematic alignment errors that produce false positive fusion boundary predictions. We expect a true positive to produce a certain number of reads split approximately in half by the fusion boundary, whereas many false positives are identified by the lack of any reads that are split approximately in half. We calculate two statistics in order to identify false positive split alignments.

For each of the n split alignments supporting a prediction, let l_i and r_i be the number of nucleotides aligning to the left and right of the fusion boundary respectively. Under the null hypothesis that the fusion boundary is real, the normalized split position p_i (equation 10), and normalized minimum split anchor q_i (equation 11) should be uniformly distributed on $[0, 1]$ and have expected value $E[p_i] = E[q_i] = 0.5$ and variance $Var[p_i] = Var[q_i] = \frac{1}{12n}$.

$$p_i = \frac{l_i - n_{anchor}}{l_i + r_i - 2n_{anchor}} \tag{10}$$

$$q_i = \frac{\min(l_i, r_i) - n_{anchor}}{\frac{l_i+r_i}{2} - n_{anchor}} \tag{11}$$

A dependence between p_i values for reads split by the same fusion boundary means that the sample variance of a set of n p_i values includes a covariance term. The covariance term and sample variance of n p_i values are calculated as described in 4.6. A dependence between q_i is resolved similarly. The samples means of the n p_i and n q_i values are assumed normally distributed.

A two sided z-test with alternative hypothesis $E[p] \neq 0.5$ is used to calculate the *split position p-value*. A one sided z-test with alternative hypothesis that $E[q] < 0.5$ is used to calculate the *minimum split anchor p-value*. Significant values for these p-values represents evidence to reject the null hypothesis that the split reads are uniformly distributed across the fusion boundary.

4.7.3 Fusion boundary di-nucleotide entropy

A common source of false positive fusion boundary predictions using split alignments results from the alignment of low complexity reads such as poly-A reads to low complexity regions in genes. In order to identify spurious fusion boundary predictions caused by low complexity reads, we calculate the di-nucldeotide entropy of the predicted fusion boundary sequence. Let $D = \{n_i n_j : n_i, n_j \in \{A, C, T, G\}\}$ be the set of all possible di-nucldeotides. Let S be a sequence of length m and let $\text{count}(d, S)$ be the number of occurrences of di-nucleotide d in sequence S. The di-nucleotide entropy of the sequence S can be calculated as given by equation 12.

$$\begin{aligned} H(S) &= -\sum_{d \in D} p_{d,S} \log_2 p_{d,S} \\ p_{d,S} &= \frac{\text{count}(d, S)}{m-1} \end{aligned} \tag{12}$$

Let S_u be the 40 nucleotides of the predicted sequence upstream of the fusion boundary, and let S_d be the 40 nucleotides of the predicted sequence downstream of the fusion boundary. For the purposes of this study we use $m = 40$. We calculate the *fusion boundary di-nucleotide entropy* as $\min(H(S_u), H(S_d))$. The fusion boundary di-nucleotide entropy is expected to be lower for fusion boundary predictions involving low complexity sequence on either side of the fusion boundary

4.7.4 Fusion boundary homology

Reverse transcriptase (RT) during cDNA preparation has been identified previously as a mechanism for producing chimeric cDNA fragments [3]. An identifying feature of chimeric cDNA produced by template switching is the existence of short homologous sequence at the 'splice site' implied by the cDNA sequence [3]. Thus, to identify predictions resulting from chimeric reads produced by template switching during RT, we calculate the length of homologous sequence at the fusion boundary.

Let S be the predicted sequence for a fusion prediction between gene X and gene Y, and let l be length of S. Let m_X and m_Y be the number of matches minus mismatches for the best alignments of S to all splice variants of X and Y respectively. We calculate an estimate of the fusion boundary homology as given by equation 13.

$$\textit{Fusion boundary homology} = m_X + m_Y - l \tag{13}$$

Note that if a prediction is caused by misalignments of non-chimeric reads from a single gene, the predicted sequence may align with high sequence similarity to only that gene. This situation will also produce a higher than normal value for the fusion boundary homology, also indicating a likely false positive. All alignments of S to splice variants of X and Y were obtained using blat [4].

4.7.5 Adjusted percent identity

We sought to identify concordant alignments of the predicted fusion sequence to cDNA, EST and chromosome sequences. However, some predicted fusion sequences are asymmetrical:

they involve only a small amount of sequence from one of the genes predicted as fused. As a result, reporting a simple percent identify for the alignment of the predicted sequence to a cDNA, EST, or chromosome would be biased against asymmetrical fusion prediction sequences. We use the *adjusted percent identity*, described below, as an alternative to the percent identity that does not suffer from a bias against asymmetrical fusion prediction sequences.

Let S be the predicted sequence for a fusion prediction between gene X and gene Y, let ζ be fusion boundary in S, and let l be the length of S. Also let S_X and S_Y be the sequences on the X and Y sides of ζ respectively, with lengths l_X and l_Y respectively. Given an alignment of S to a cDNA, EST or chomosome sequence, let m be the matches minus mismatches for the alignment. We first assume that the longer of S_X and S_Y is matched exactly in the alignment, and any remaining matches exist in the shorter of S_X and S_Y. We then calculate the *adjusted percent identity* as the percent identity of the alignment within the shorter of S_X and S_Y under these assumptions (equation 14). All alignments of S to cDNA, EST and chromosome sequences were obtained using blat [4].

$$\textit{Adjusted percent identity} = \frac{m - \max(l_X, l_Y)}{\min(l_X, l_Y)} \tag{14}$$

4.7.6 EST islands

We sought to identify predictions that could be explained by alternative splicing as opposed to underlying genomic structural variation. We use UCSC's spliced EST alignments [6] as evidence of co-transcription of genomic regions. An EST island is then defined as the set of minimal genomic regions such that any splice EST alignment that overlaps with an EST island is contained within that EST island. EST islands represent islands of co-transcription in the genome as evident by EST alignments. The *EST island adjusted percent identity* for a fusion prediction is the *adjusted percent identity* of a spliced alignments of the predicted sequence that falls entirely within an EST island.

4.8 Filtering

A principled machine learning approach to discriminating between true and false positives is difficult without a significant number of positive and negative controls. Thus in order to roughly discriminate between real fusions and false positives, we initially used a set of thresholds on a subset of the features calculated in section 4.7. These thresholds are given below.

Spanning read count	$>$	5
Split read count	$>$	3
Spanning read coverage	$>$	0.6
Split position p-value	$>$	0.1
Minimum split anchor p-value	$>$	0.1
Corroboration p-value	$>$	0.1
Concordant ratio	$<$	0.1
cDNA adjusted percent identity	$<$	0.1
Genome adjusted percent identity	$<$	0.1
EST adjusted percent identity	$<$	0.3
EST island adjusted percent identity	$<$	0.3

4.9 Probabilistic motivation for clustering conditions

The two conditions for clustering paired end alignments can be motivated probabilistically by considering the likelihood of two paired end alignments given that those paired end reads represent the same fusion transcript. Consider the alignments of two discordant paired end reads, a and b. Suppose a has an alignment of end a_X to transcript X and end a_Y to transcript Y. Similarly, suppose b has an alignment of end b_X to transcript X and an alignment of end b_Y to transcript Y. Figure 3 shows a possible configuration of the alignments.

The distances d_X and d_Y are the differences between the positions of alignments on transcript X and transcript Y respectively. Also, v is the latent variable representing the unknown

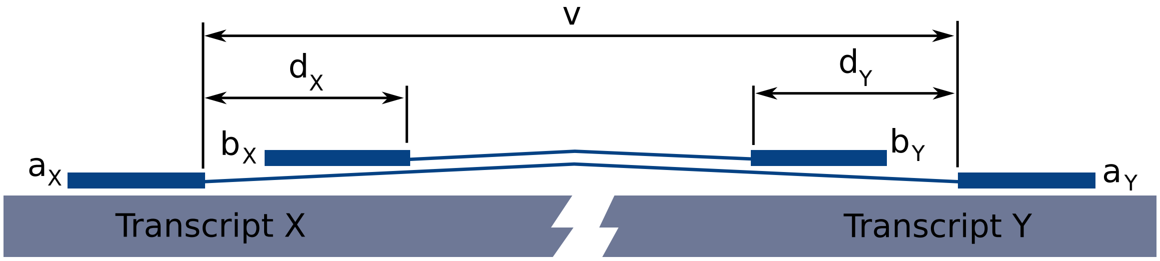

Figure 3: Paired end configuration

length of the unsequenced region of paired end a. Given v, we can calculate the fragment lengths x_a and x_b of paired end reads a and b as,

$$\begin{aligned} x_a &= v + 2r \\ x_b &= v - d_X - d_Y + 2r, \end{aligned}$$

where r is the read length.

Thus given v and supposing that paired end reads a and b result from the same fusion isoform F, we can calculate the probability $P(d_X, d_Y|v, F)$ as

$$P(d_X, d_Y|v, F) = \begin{cases} \mathcal{N}(x_a|\mu, \sigma)\mathcal{N}(x_b|\mu, \sigma) & \text{for } v \geq d_X + d_Y\,, \\ 0 & \text{otherwise}\,, \end{cases}$$

where μ and σ are the inferred fragment length mean and standard deviation. We can now use the fact that $P(d_X, d_Y, v|F) \propto P(d_X, d_Y|v, F)$ to calculate $P(d_X, d_Y|F)$.

$$\begin{aligned} P(d_X, d_Y|F) &= \sum_v P(d_X, d_Y, v|F) \\ &= \frac{1}{Z} \sum_v P(d_X, d_Y|v, F) \end{aligned}$$

Z is a normalization constant calculated as follows.

$$Z = \sum_{v} \sum_{d_X} \sum_{d_Y} P(d_X, d_Y | v, F)$$

Figure 4 shows the probability distribution $P(d_X, d_Y|F)$ for $r = 50$, $\mu = 200$ and $\sigma = 30$.

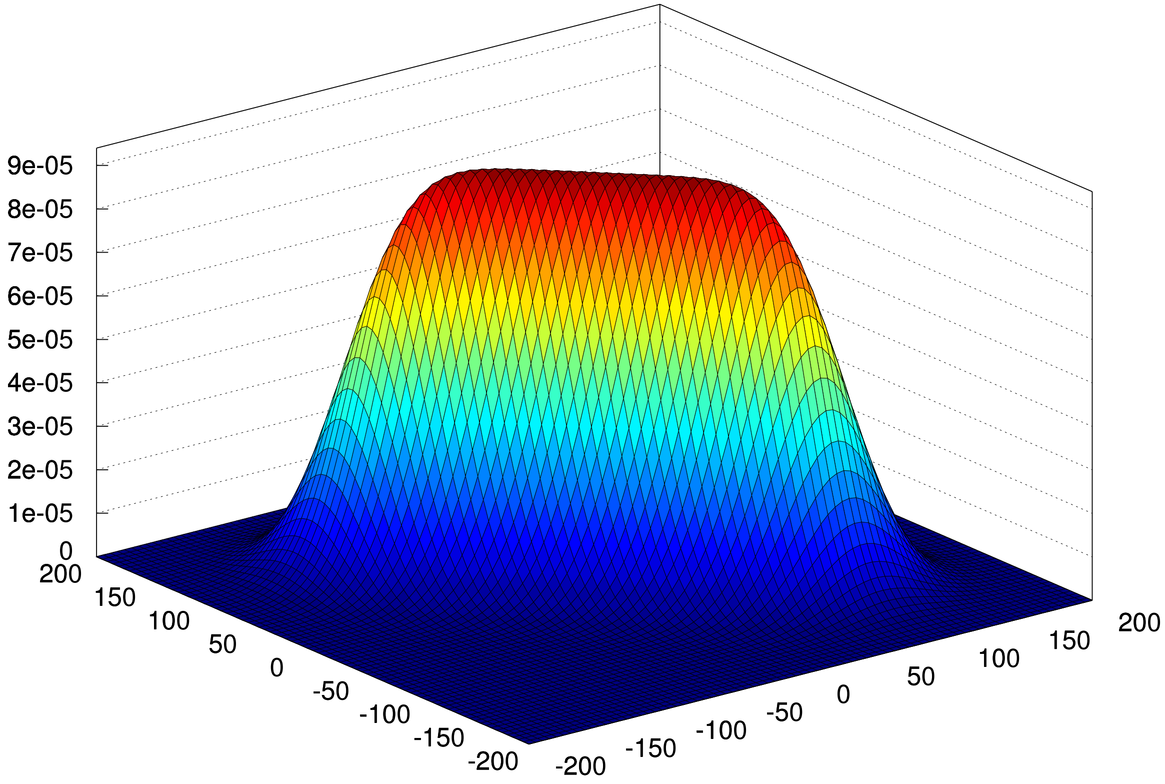

Figure 4: Probability distribution $P(d_X, d_Y|F)$

The overlapping boundary region condition and the similar fragment length condition have equivalent formulations as constraints on d_X and d_Y. The overlapping boundary region condition is equivalent to the constraints given by equations 15 and 16. Any values for d_X or d_Y outside these constraints will result in non overlapping fusion boundary regions for transcript X or transcript Y. Values for d_X and d_Y that satisfy constraints given by both equations

15 and 16 will have overlapping boundary regions and will satisfy the overlapping boundary region condition. The similar fragment length condition is equivalent to the constraint $-(l_{max} - l_{min}) \leq d_X + d_Y \leq l_{max} - l_{min}$, which is simply a reformulation of the equation in figure 2c.

$$
\begin{aligned}
-l_{min} - 2r < \quad & d_X & < l_{max} + 2r \qquad (15) \\
-l_{min} - 2r < \quad & d_Y & < l_{max} + 2r \qquad (16) \\
-(l_{max} - l_{min}) \leq \quad & d_X + d_Y & \leq l_{max} - l_{min} \qquad (17)
\end{aligned}
$$

We compared the region of the $d_x \times d_y$ configuration space that satisfies the overlapping boundary region condition and similar fragment length condition for $\alpha = 0.05$ with a region contained within an equivalent contour of $P(d_X, d_Y|F)$. We used $r = 50$, $\mu = 200$ and $\sigma = 30$ as was used for figure 4. We calculated $l_{max} - l_{min}$ for $\alpha = 0.05$ and then calculated $q = \sum_{|l_i - l_j| < l_{max} - l_{min}} P(l_i)P(l_j) = 0.99422$. The value q represents the combined probablity of seeing two fragments that satisfy constraints given by equations 15, 16, and 17 for $\alpha = 0.05$. We then calculated the contour of $P(d_X, d_Y|F)$ that contains probability mass equal to q. Figure 5 shows the region of the configuration space satisfying the two conditions together with the equivalent contour of $P(d_X, d_Y|F)$.

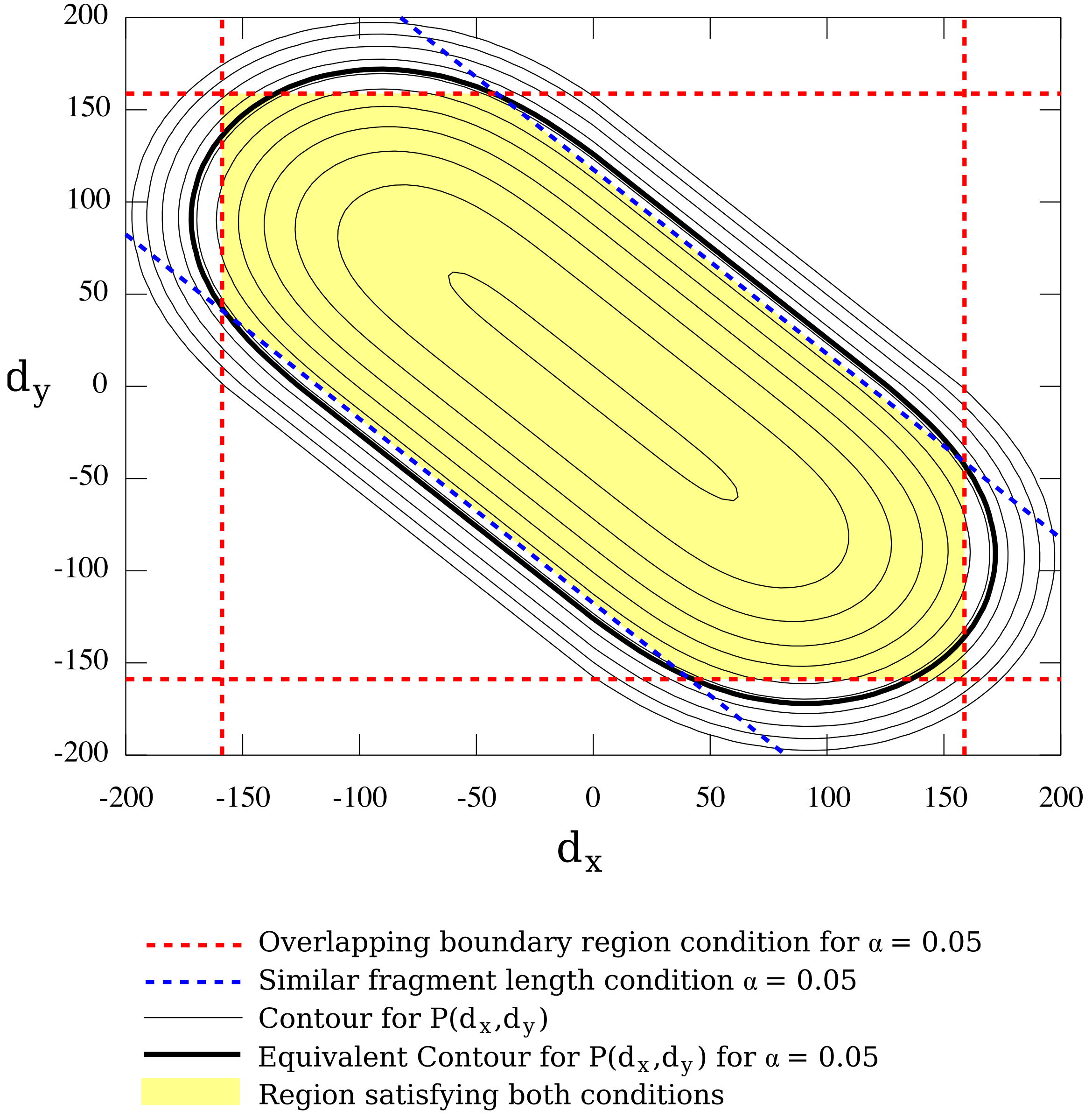

Figure 5: Overlapping boundary region condition and similar fragment length condition in the context of probability distribution $P(d_X, d_Y|F)$. For $\alpha = 0.05$, the region of the configuration space satisfying the two conditions overlaps with the equivalent contour of $P(d_X, d_Y|F)$.

4.10 FusionSeq predictions

FusionSeq version 0.6.1 was used to predict gene fusions in CCC15, CCC16 and EMD6. These 3 cases were chosen because they contained the greatest number of validated predictions, with 6, 3 and 4 validations respectively. We followed the instructions provided on the FusionSeq and RSeqTools websites, reiterated here. We first downloaded the hg18 bundled dataset. We then created a junction library from the ucsc provided 2bit genome and the gene models provided in the bundled dataset using the following command:

```
createSpliceJunctionLibrary hg18.2bit knownGeneAnnotationTranscriptCompositeModel.txt 45
```

Next we used `bowtie-build` to generate bowtie indices for the human genome and junction library combined. Bowtie was used with default parameters to independently generate alignments for each end of the paired end reads. The two bowtie outputs were converted into MRF format using the `bowtiePairedFix` executable provided by the author and `bowtie2mrf` from RSeqTools.

```
bowtie hg18_junctions reads.1.fastq reads.1.bwtout
bowtie hg18_junctions reads.2.fastq reads.2.bwtout
cat reads.1.bwtout reads.2.bwtout | sort | bowtiePairedFix | bowtie2mrf paired -sequence > data.mrf
```

Fusions were predicted based on the data.mrf file using the following commands with default parameters as given by the FusionSeq website:

```
geneFusions data 4 < data.mrf > data.1.gfr 2> data.1.log
(gfrAbnormalInsertSizeFilter 0.01 < data.1.gfr | gfrPCRFilter 4 4 | gfrProximityFilter 1000
   | gfrAddInfo | gfrAnnotationConsistencyFilter ribosomal | gfrLargeScaleHomologyFilter
   | gfrRibosomalFilter | gfrSmallScaleHomologyFilter) > data.gfr 2> data.log
gfrConfidenceValues data < data.gfr > data.confidence.gfr
```

To compare the overlap between FusionSeq predictions and deFuse predictions, we aligned the FusionSeq read evidence to fusion sequences predicted by deFuse using bowtie. Comparing the results in this way avoided problems that would result from trying to compare gene identifiers from different sets of gene annotations.

We also sought to validate fusions predicted by FusionSeq that were not predicted by deFuse. In order to maximize our chances of successful validation, we applied a set of filters to the FusionSeq output before selecting fusions to validate. We first sought to classify as concordant reads that were evidence for the FusionSeq predictions. We aligned the read evidence to the genome and ESTs from UCSC, and searched for alignments of each within 1000nt of

each other on the same chromosome/EST. We removed any FusionSeq prediction for which at least one read could be classified as concordant using this method. We also removed FusionSeq predictions for which at least one end of one read aligned to a ribosomal RNA (ensembl 54 gene models). Since we were were not interested in differences between the results that arose because of the use of different sets of gene annotations, we removed FusionSeq predictions for which none of the reads aligned using blat to gene regions considered by deFuse. Several of the predictions were removed because they involved reads that did not align to a contiguous region of the genome or to contiguous exons, making it difficult to pinpoint a breakpoint and design primers. Finally, we removed fusions also predicted by deFuse, and selected the 3 predictions from each library with the highest RESPER score. This produced 3 candidates for CCC16 and EMD6, and 2 candidates for CCC16 which only contained 2 fusions after filtering.

4.11 MapSplice predictions

MapSplice version 1.14.1 was first used predict fusions in CCC15, CCC16 and EMD6. To reiterate, these 3 cases were chosen because they contained the greatest number of validated predictions, with 6, 3 and 4 validations respectively. We followed the set of instructions on the MapSplice website, downloading the ucsc genome and building a bowtie index. The default paired end configuration file was used, with the following differences.

```
read_length = 50
segment_length = 16
junction_type = non-canonical
run_MapPER = yes
full_running = no
do_fusion = yes
```

We then searched the MapSplice results for the validated deFuse predictions. We selected all of the sequences in the *synthetic sequence* column of `fusion.junction` file and used blat with default parameters to find an alignment of those junction sequences to the sequences predicted by deFuse. We also extracted all splice junction predictions from the CIGAR string of each alignment in the `alignments.sam` file, and compared those splice junction predictions with the validated deFuse predictions.

We suspected MapSplice might perform better on the 75mer libraries. Thus, we ran MapSplice on the 75mer reads from SCH1, EMD5 and GRC5. The default paired end configuration file was used, with the following differences.

```
read_length = 75
segment_length = 25
junction_type = non-canonical
run_MapPER = yes
do_fusion = yes
```

We sought to validate fusions predicted by MapSplice that were not predicted by deFuse. In order to maximize our chances of successful validation, we applied a set of conservative filters to the MapSplice output before selecting fusions to validate. From the `fusion.junction` file we selected fusions with at least 2 supporting reads that were predicted to occur within the boundaries fo the ensembl genes we were considering in this study. We then removed predictions for which the *synthetic sequence* aligned with greater than 90% identity by blat to the genome, or greater than 50% identity to ribosomal RNA. After applying these filters we were left with 14 predictions from CCC15, CCC16, EMD6, SCH1, EMD5 and GRC5.

4.12 Running deFuse on melanoma RNA-Seq datasets

RNA-Seq datasets for 13 melanoma samples and cell lines were downloaded from the short read archive. These datasets are half the size of our average sarcoma or ovarian cancer datasets, and 4 of the fusions represented in these datasets have 5 or less supporting reads. Thus we adjusted the following parameters of deFuse so that deFuse would be able to predict fusions in these datasets. The `clustering_precision` parameter is equal to $1 - \alpha$.

```
clustering_precision = 0.80
span_count_threshold = 2
split_count_threshold = 1
```

4.13 Calculating expression from RNA-Seq alignments

Reads were aligned to the genome (NCBI36/hg 18) using MAQ (0.7.1) [5] and allowing for up to 5 mismatches. Raw expression values (read counts) were obtained by summing the number of reads that mapped to human genes based on the Ensembl database (Release 51). Gene expression values were normalized using a quantile normalization procedure using aroma.light (1.16.0.) package in R (2.11.1).

4.14 Inferring copy number from Affymetrix SNP 6.0

The Affymetrix SNP6.0 arrays were normalized using CRMAv2 [1] using the default settings for performing allelic-crosstalk calibration, probe sequence effects normalization, probe-level summarization, and PCR fragment length normalization. Log ratios are then computed by normalizing against a pooled reference generated using a normal dataset of 270 HapMap samples obtained from Affymetrix. Segmentation is performed using a modified version of a hidden Markov model for detecting aCGH copy number, CNA-HMM [7]. The model has been extended to analyze high-density genotyping array platforms (available for download at http://compbio.bccrc.ca/) . The HMM model performs segmentation of the log ratio intensity data and predicts discrete copy number status for each resulting segment from the set of 6 possible states (homozygous deletion, hemizygous deletion, neutral, gain, amplification, and high-level amplifcation). The boundaries of the segments provide candidate breakpoints in the genome as a result of copy number alteration events. CNV predictions are provided in supplementary table S3. The given data is formated to be visualized using IGV (http://www.broadinstitute.org/igv).

5 Supplementary Experimental Methods

5.1 Paired-End RNA Sequencing

For each patient sample polyadenylated RNA was purified from 10ug of DNAse1 (Invitrogen, Carlsbad, CA) treated total RNA using the MACSTM mRNA Isolation Kit (Miltenyi Biotec, Germany). Double-stranded cDNA was synthesized from the purified polyA+RNA using Superscript^{TM}. Double-Stranded cDNA Synthesis kit (Invitrogen, Carlsbad, CA) and random hexamer primers (Invitrogen) at a concentration of 5M. The resulting cDNA was sheared using a Sonic Dismembrator 550 (Fisher Scientific, Canada) and size separated by PAGE (8%). The 190-210bp DNA fraction was excised, eluted overnight at 4C in 300 uL of elution buffer (5:1, LoTE buffer (3 mM Tris-HCl, pH 7.5, 0.2 mM EDTA)-7.5 M ammonium acetate) and purified using a QIAquick purification kit (Qiagen, Mississauga, ON). The sequencing library was prepared following the Illumina Genome Analyzer paired end library protocol (Illumina Inc., Hayward, CA) with 10 cycles of PCR amplification. PCR products were purified on Qiaquick MinElute columns (Qiagen, Mississauga, ON) and assessed and quantified using an Agilent DNA 1000 series II assay and Qubit fluorometer (Invitrogen,

Carlsbad, CA) respectively. The resulting libraries were sequenced on an Illumina Genome Analyzer II following the manufacturer's instructions. Sequencing read lengths varied between 36 and 75 nucleotides. Image analysis and basecalling was performed by the GA pipeline v1.0 (Illumina Inc., Hayward, CA) using phasing and matrix values calculated from a control phiX174 library run on each flowcell. Raw Quality scores were calibrated by alignment to the reference human genome (NCBI build 36.1, hg18) using ELAND (Illumina Inc., Hayward, CA).

5.2 Direct sequencing

RNA was extracted from frozen tumors using Qiazol (Qiagen, Valencia, CA) and reverse transcribed using SuperScriptIII (Invitrogen, Carlsbad, CA). The cDNA was amplified using the primers as given in supplementary table S5 using PCR SuperMix High Fidelity (Invitrogen, Carlsbad, CA). The cycling parameters were: an initial denaturation of 94C for 1 min. followed by 35 cycles of 94C 30sec denaturation, 58C 30sec annealing and 72C 30sec extension, followed by a final extension of 72C for 5min. PCR was performed on a MJ Research Tetrad (Ramsey, MN). PCR products were purified using a MinElute PCR purification kit (Qiagen, Valencia, CA) and bi-directionally sequenced using an ABI BigDye terminator v3.1 cycle sequencing kit (Applied Biosystems, Foster City, CA) and an ABI Prism 3130xl Genetic Analyzer (Applied Biosystems, Foster City, CA). Sequence traces in ab1 format are provided in supplementary data S9.

5.3 Fluorescent in situ hybridization

Metaphases and metaphase slides were produced by using standard methods. Locus-specific FISH analysis was performed by using BACs from Human BAC library RPC1-11 (BACPAC Resources Centre, Childrens Hospital, Oakland Research Institute). Supplementary table S4 shows the locations of the BAC probes used for gene fusion validation. BACs were directly labeled with either Spectrum green or Spectrum orange (Vysis, Downer's Grove, IL). The chromosomal locations of all BACs were validated by using normal metaphases (results not shown). Probe labeling and FISH was performed by using Vysis reagents according to the manufacturer's protocols. Slides were counterstained with DAPI for microscopy. For all slides, FISH signals and patterns were identified on a Zeiss Axioplan epifluorescent microscope. Signals were interpreted manually, and images were captured by using the ISIS

FISH imaging software (MetaSystems Group, Belmont, MA). A cutoff of 2 breaks per 100 nuclei was selected for a positive score based on examining 230 other soft-tissue tumors. Efficiency of the break-apart FISH probes on TMAs was demonstrated with the t(X;18) in synovial sarcomas [9]. FISH images are provided in supplementary data S10.

References

[1] H Bengtsson, P Wirapati, and T P Speed. A single-array preprocessing method for estimating full-resolution raw copy numbers from all Affymetrix genotyping arrays including GenomeWideSNP 5 & 6. *Bioinformatics*, 25(17):2149–2156, Sep 2009.

[2] F Hormozdiari, C Alkan, E E Eichler, and S C Sahinalp. Combinatorial algorithms for structural variation detection in high-throughput sequenced genomes. *Genome Res*, 19(7):1270–1278, Jul 2009.

[3] J Houseley and D Tollervey. Apparent non-canonical trans-splicing is generated by reverse transcriptase in vitro. *PLoS One*, 5(8), 2010.

[4] W James Kent. BLAT–the BLAST-like alignment tool. *Genome Res*, 12(4):656–64, Apr 2002.

[5] H Li, J Ruan, and R Durbin. Mapping short DNA sequencing reads and calling variants using mapping quality scores. *Genome Res*, 18(11):1851–1858, Nov 2008.

[6] B Rhead, D Karolchik, R M Kuhn, A S Hinrichs, A S Zweig, P A Fujita, M Diekhans, K E Smith, K R Rosenbloom, B J Raney, A Pohl, M Pheasant, L R Meyer, K Learned, F Hsu, J Hillman-Jackson, R A Harte, B Giardine, T R Dreszer, H Clawson, G P Barber, D Haussler, and W J Kent. The UCSC Genome Browser database: update 2010. *Nucleic Acids Res*, 38(Database issue):613–619, Jan 2010.

[7] S P Shah, X Xuan, R J DeLeeuw, M Khojasteh, W L Lam, R Ng, and K P Murphy. Integrating copy number polymorphisms into array CGH analysis using a robust HMM. *Bioinformatics*, 22(14):431–439, Jul 2006.

[8] T F Smith and M S Waterman. Identification of common molecular subsequences. *J Mol Biol*, 147(1):195–7, Mar 1981.

[9] J Terry, T S Barry, D E Horsman, F D Hsu, A M Gown, D G Huntsman, and T O Nielsen. Fluorescence in situ hybridization for the detection of t(X;18)(p11.2;q11.2) in a synovial sarcoma tissue microarray using a breakapart-style probe. *Diagn Mol Pathol*, 14(2):77–82, Jun 2005.
